# Cytokine-Mediated Alterations of Human Cardiac Fibroblast’s Secretome

**DOI:** 10.3390/ijms222212262

**Published:** 2021-11-12

**Authors:** Hanna Bräuninger, Tilo Thottakara, Jacob Schön, Svenja Voss, Vishnu Dhople, Svenja Warnke, Katharina Scherschel, Benedikt Schrage, Paulus Kirchhof, Stefan Blankenberg, Uwe Völker, Dirk Westermann, Elke Hammer, Diana Lindner

**Affiliations:** 1 Department of Cardiology, University Heart & Vascular Centre, University Hospital Hamburg-Eppendorf, Martinistrasse 52, 20246 Hamburg, Germany; h.braeuninger@uke.de (H.B.); t.thottakara@uke.de (T.T.); Svenja.voss@yahoo.com (S.V.); s.warnke@uke.de (S.W.); katharina.scherschel@evk-duesseldorf.de (K.S.); b.schrage@uke.de (B.S.); p.kirchhof@uke.de (P.K.); s.blankenberg@uke.de (S.B.); d.westermann@uke.de (D.W.); 2 DZHK (German Centre for Cardiovascular Research), Partner Site Hamburg/Kiel/Lübeck, 20246 Hamburg, Germany; 3 Department of Functional Genomics, Interfaculty Institute for Genetics and Functional Genomics, University Medicine Greifswald, Felix-Hausdorff-Str. 8, 17475 Greifswald, Germany; jacob.schoen@fli.de (J.S.); dhoplevm@uni-greifswald.de (V.D.); voelker@uni-greifswald.de (U.V.); hammer@uni-greifswald.de (E.H.); 4 DZHK (German Centre for Cardiovascular Research), Partner Site Greifswald, 17475 Greifswald, Germany; 5 Division of Cardiology (cNEP), EVK, 40217 Düsseldorf, Germany; 6 Medical Faculty, Institute of Neural and Sensory Physiology, Heinrich Heine University Düsseldorf, 40225 Düsseldorf, Germany; 7 Institute of Cardiovascular Sciences, University of Birmingham, Birmingham B15 2TT, UK

**Keywords:** cytokine, cardiac fibroblast, TGF-β, TNF-α, secretome, proteome, transcriptome, mass spectrometry, Affymetrix

## Abstract

Fibroblasts contribute to approximately 20% of the non-cardiomyocytic cells in the heart. They play important roles in the myocardial adaption to stretch, inflammation, and other pathophysiological conditions. Fibroblasts are a major source of extracellular matrix (ECM) proteins whose production is regulated by cytokines, such as TNF-α or TGF-β. The resulting myocardial fibrosis is a hallmark of pathological remodeling in dilated cardiomyopathy (DCM). Therefore, in the present study, the secretome and corresponding transcriptome of human cardiac fibroblasts from patients with DCM was investigated under normal conditions and after TNF-α or TGF-β stimulation. Secreted proteins were quantified via mass spectrometry and expression of genes coding for secreted proteins was analyzed via Affymetrix Transcriptome Profiling. Thus, we provide comprehensive proteome and transcriptome data on the human cardiac fibroblast’s secretome. In the secretome of quiescent fibroblasts, 58% of the protein amount belonged to the ECM fraction. Interestingly, cytokines were responsible for 5% of the total protein amount in the secretome and up to 10% in the corresponding transcriptome. Furthermore, cytokine gene expression and secretion were upregulated upon TNF-α stimulation, while collagen secretion levels were elevated after TGF-β treatment. These results suggest that myocardial fibroblasts contribute to pro-fibrotic and to inflammatory processes in response to extracellular stimuli.

## 1. Introduction

Cardiac fibroblasts represent approximately 20% of the non-cardiomyocytic cells in the heart [1,2]. The main physiological function of cardiac fibroblasts is the maintenance and regulation of myocardial stiffness and protection against dilation by the production of the extracellular matrix (ECM) [3]. In addition to regulating ECM homeostasis in normal myocardial function, fibroblast activation is an important component of the adaptation of the heart to stressors such as viral infection, myocardial infarction, or heart failure.

Myocardial fibrosis is an important predictor of ventricular arrhythmias, deterioration of cardiac function, and cardiovascular mortality [4,5,6]. Excessive deposition of ECM proteins by cardiac fibroblasts plays a pivotal role in the pathological processes of many cardiac diseases, such as dilated cardiomyopathy (DCM). DCM is defined by its two hallmarks, ventricular enlargement and systolic dysfunction [7]. Here, fibrosis is leading to reduced compliance of the cardiac wall, thereby maintaining the required stiffness of the enlarged heart but also accelerating the progression of heart failure.

In DCM’s pathophysiology, TNF-α, as well as TGF-β signaling, are involved. Accordingly, patients with heart failure exhibit increased circulating levels of TNF-α [8]. In addition, locally produced TNF-α can contribute to DCM progression by inducing negative inotropic effects through the enhancement of nitric oxide [9]. Furthermore, it was shown that TNF-α-secreting B cells were increased in blood samples of DCM patients and that TNF-α can contribute to myocardial fibrosis in DCM [10]. TGF-β regulates a well-known major pro-fibrotic pathway in the heart [1,11] that can lead to transdifferentiation of fibroblasts to myofibroblasts [12].

Even though TNF-α and TGF-β have been assumed to influence the development of myocardial fibrosis in DCM, the exact mechanism remains elusive. Since fibroblasts are the key cells for ECM maintenance, the purpose of this study was to analyze the secretome and its corresponding gene expression of human cardiac fibroblasts from DCM patients by untargeted approaches. Thus, we utilized tandem mass spectrometry and Affymetrix analysis of quiescent human cardiac fibroblasts as well as after TNF-α or TGF-β stimulation to reveal autocrine and paracrine factors, whereof some might be involved in the disease progression of DCM.

## 2. Results

### 2.1. Primary Human Cardiac Fibroblasts Isolated from Biopsies

**Figure 1:**
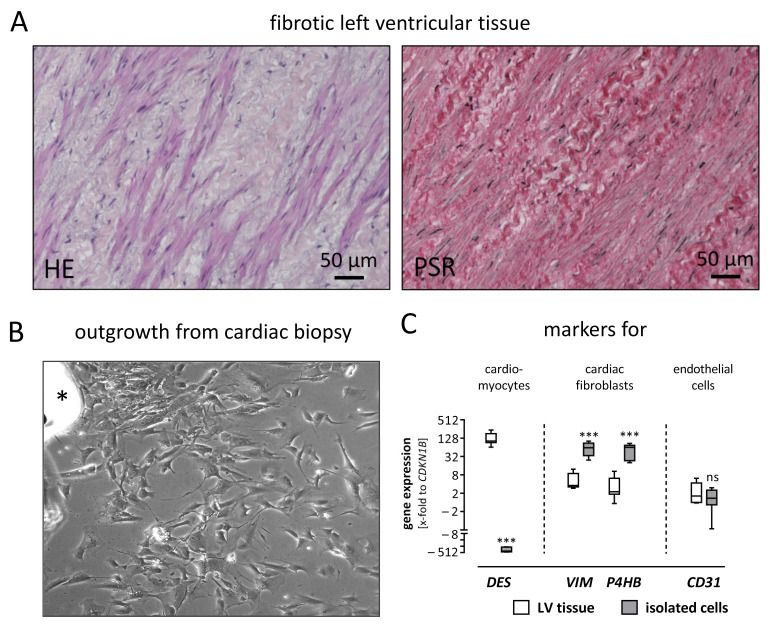
Isolated cardiac cells derived from left ventricular tissue were characterized as fibroblasts. (**A**) Left ventricular (LV) tissue sections were stained using Hematoxylin & Eosin (HE) and Pico Sirius Red (PSR). In the HE staining, spindle-shaped cells—presumably cardiac fibroblasts—are visible in the fibrotic LV tissue. The cells are embedded within thick, curled fibers, which are confirmed as collagen fibers by PSR staining. (**B**) Spindle-shaped cells are outgrowing from a cardiac biopsy (upper left side, asterisk) and spreading over the cell culture plate. (**C**) To confirm the isolated cardiac cells as fibroblasts, the gene expression of LV tissue and isolated cardiac cells were compared regarding cell-type-specific markers. In the LV tissue, the muscle marker desmin (*DES*) is higher expressed, while vimentin (*VIM*) and prolyl 4-hydroxylase (*P4HB*) as markers for fibroblasts are lower expressed, compared to the isolated cardiac cells. A difference in the expression of the endothelial marker *CD31* is not observed between LV tissue and isolated cardiac cells. For statistical comparisons, a *t*-test was used. *** *p* ≤ 0.001; ns, not significant.

In the late stage of DCM, the morphology of the left ventricular myocardium is strongly altered, as exemplarily shown in Figure 1A. Within the extensive amount of extracellular matrix proteins (stained by Pico Sirius Red), cardiac fibroblasts are clearly visible as embedded spindle-shaped cells. For this study, human cardiac fibroblasts were obtained by outgrowth utilizing cardiac biopsies from patients suffering from DCM (Figure 1B). To verify that these spindle-shaped, outgrown cells were indeed cardiac fibroblasts, gene expression of cell-type-specific markers was assessed and compared to left ventricular tissue (Figure 1C). The isolated cells revealed an increased gene expression in fibroblast-specific genes such as vimentin (*VIM*) and prolyl 4-hydroxylase (*P4HB*), whereas the expression of muscle-specific desmin (*DES*) and the endothelial marker *CD31* was reduced or unchanged, respectively.

### 2.2. Identification of Human Cardiac Fibroblast’s Secretome

Cell culture supernatant from quiescent human cardiac fibroblasts derived from four patients was analyzed utilizing label-free mass spectrometry to identify and quantify proteins in the extracellular fraction. Proteins, which were consistently identified in the cell culture supernatants of all patients, were defined as extracellular proteomes. In total, 166 extracellular proteins were detected. To identify the secretome, these 166 proteins were compared to 2091 proteins annotated as secreted (Uniprot 2021); 112 of 166 extracellular proteins (67%) were assigned as the secretome of human cardiac fibroblasts indicated by black boundaries. According to the respective Uniprot annotations, 27 proteins were assigned to membrane or endoplasmic reticulum fractions.

**Figure 2:**
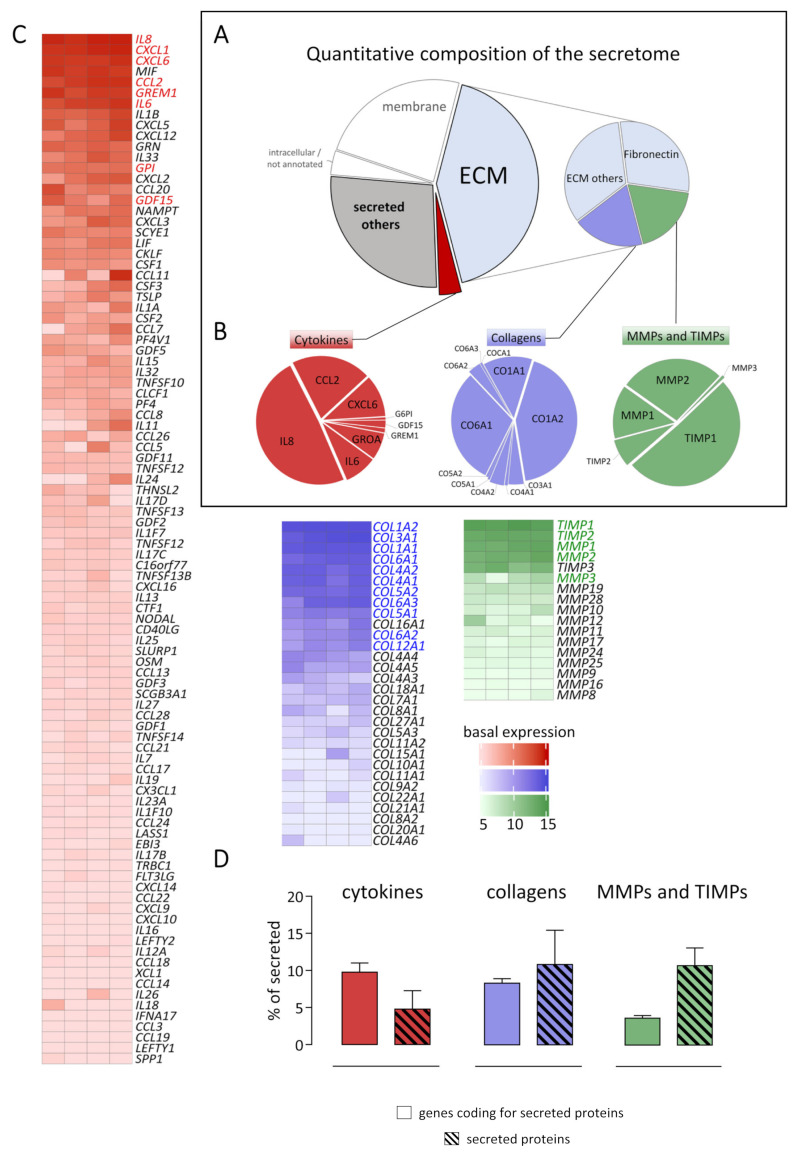
Quantitative composition of the secretome and corresponding transcriptome of human cardiac fibroblasts. (**A**) Cell culture supernatants from human cardiac fibroblasts derived from four different patients were analyzed by tandem mass spectrometry. Untreated control cells were used to analyze the composition of the extracellular proteome and to define the secretome. Proteins were categorized according to their localization annotation in Uniprot (Subcellular locations, 2021). In total, 166 proteins were identified as extracellular proteome, while 112 proteins were annotated as secreted proteins. As plotted in the pie chart, these secreted proteins represent 72% of the total proteome amount (intensity). The secretome is highlighted with black boundaries. The protein intensities of each detected protein is listed in Supplemental Table S1. (**B**) The quantitative composition of the three subcategories cytokines, collagens, as well as MMPs/TIMPs were analyzed in more detail. (**C**) Total RNA isolated from human cardiac fibroblasts derived from four different patients was analyzed using the human genome U133 Plus 2.0 Array (Affymetrix). The expression of genes of the three subcategories was analyzed in untreated control cells, and log_2_ transformed expression data are visualized as heatmaps. For genes with more than one Affymetrix ID, the highest signal was plotted. The raw data for each gene ID is listed in alphabetical order in Supplemental Tables S2–S4. (**D**) The intensities of the categories cytokines (red), collagens (blue), and MMPs/TIMPs (green) were compared on protein and gene expression levels in relation to all secreted proteins or all genes coding for secreted proteins, respectively.

To quantify the amount of the identified secreted proteins, intensity-based absolute quantification values (iBAQ) were calculated. The 112 secreted proteins represent 72% of the protein amount, as shown in Figure 2A. Subsequently, the secretome was further divided into three groups: (1) ECM, extracellular matrix; (2) cytokines, and (3) other secreted proteins. All categories were assigned respectively to their Uniprot Annotation as described in the material and methods section. Out of the secretome, cytokines represent the smallest fraction (5% of total secretome), while the ECM fraction is the largest (58% of total secretome). Therefore, this fraction was further subdivided into four categories. Fibronectin represented the main part (29% of ECM fraction and 17% of total secretome), whereas collagens (blue) and matrix metalloproteinases / tissue inhibitors of matrix metalloproteinases (MMPs/TIMPs, green) accounted for 19% of ECM fraction and 11% of the total secretome.

In Figure 2B, all individual proteins assigned to the categories cytokines (red), collagens (blue), or MMPs/TIMPs (green) are displayed. Those categories were subsequently analyzed on the gene expression level utilizing the human genome U133 Plus 2.0 Array (Affymetrix). Out of all detected genes, all genes coding for secreted proteins according to the Uniprot annotation (1176 genes) were compared to the protein abundance in the secretome (112 proteins). As indicated in Figure 2B,C, the most abundant cytokine IL8 in the secretome was also highest expressed on gene expression level (red pie chart and heatmap). Out of the five highest expressed cytokines on gene expression level, four were detected in the secretome. In the case of collagens (blue), the most abundant collagen on protein level CO1A2 was most highly expressed on gene expression level. Despite different descending orders between the secretome and transcriptome of secreted proteins, the most abundant genes and proteins were similar. TIMP1 was the highest expressed candidate in the category of MMPs/TIMPs on gene expression and protein level. While *TIMP3* mRNA was highly expressed, the corresponding protein was not detected. However, despite small differences, a significant correlation between gene expression and protein level in the secretome was observed not only in the three inspected subcategories but also across all detected proteins (Supplemental Figure S1). While 96 cytokines were expressed on the gene expression level, only 8 corresponding secreted proteins were detected by mass spectrometry. This is in line with the finding that cytokines represent 10% intensity of the transcriptome of secreted proteins but only 5% of the secretome on protein level, as displayed in Figure 2D. In contrast, the percentage of secreted collagens was not different between the secretome and corresponding transcriptome. However, MMPs/TIMPs represented a larger fraction on protein (11% of total secretome) than on gene expression level (4% of the transcriptome of secreted proteins).

### 2.3. Alteration of the Fibroblast’s Secretome after Treatment with TNF-α and TGF-β

Human cardiac fibroblasts were treated with TNF-α or TGF-β, to investigate alterations of the identified secretome in response to the respective cytokines. After six hours of treatment, cells were studied by fluorescence microscopy. Gene expression analysis was carried out after 24 h of treatment, while the cell culture supernatant was collected for mass spectrometry after 72 h of treatment.

**Figure 3:**
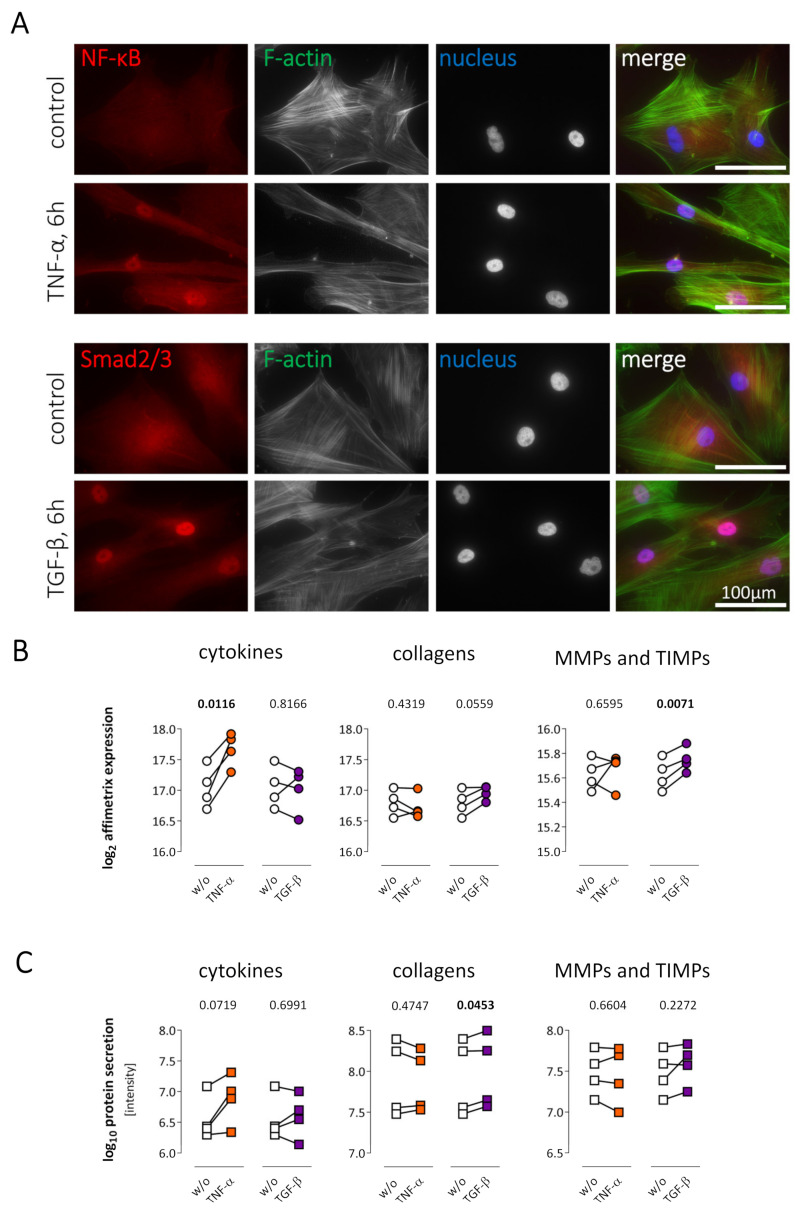
Stimulation of human cardiac fibroblasts by TNF-α or TGF-β. (**A**) A cytoplasm-to-nucleus translocation of NF-κB (red, upper panel) or SMAD2/3 (red, lower panel) was detected 6 h after TNF-α or TGF-β treatment, respectively. F-actin (green) visualizes the cell shape, while nuclei are stained with Hoechst (blue). As shown in red, diffuse cytoplasmic staining for NF-κB and SMAD2/3 was detected in untreated control cells, whereas translocation into the nucleus was clearly detectable in the co-localization with Hoechst staining after cytokine treatment. (**B**) Human cardiac fibroblasts (n = 4 patients) were treated with TNF-α (orange) or TGF-β (purple) for 24 h followed by Affymetrix analysis. Summed gene expression of genes coding for secreted proteins, as well as expression of the three subcategories cytokines, collagens, and MMPs/TIMPs is displayed as log_2_-transformed data. TNF-α induced an increased cytokine expression and TGF-β an elevated TIMP/MMP expression. (**C**) Human cardiac fibroblasts (n = 4 patients) were treated with TNF-α (orange) or TGF-β (purple) for 72 h followed by mass spectrometric analysis of the secretome. The sum of all protein intensities for each of the three subcategories cytokines, collagens, and MMPs/TIMPs were plotted as log_10_ transformed data to visualize their quantity. Fold changes of all secreted proteins after TNF-α and TGF-β stimulation are available in Supplemental Table S5. For statistical analyses, paired *t*-test was used. Statistically significant results (*p* < 0.05) are highlighted in bold. ns, not significant; w/o, untreated controls.

TNF-α or TGF-β stimulation mediated cytoplasm-to-nucleus translocation of the transcription factors NF-κB or SMAD2/3, respectively. The nuclear accumulation is clearly detectable, as shown in Figure 3A. Furthermore, Affymetrix analysis did reveal an increase in cytokine gene expression after TNF-α treatment, while cytokine gene expression was not altered after TGF-β stimulation (Figure 3B). In contrast, after TGF-β treatment, a significant increase in expression of MMPs/TIMPs was observed. TNF-α stimulation did alter neither collagen nor MMP/TIMP gene expression. An increased cytokine secretion following TNF-α stimulation was not observed on the secretome level, indicating that that the regulation observed on the transcriptomic level did not change the overall cytokine content within the secretome. In contrast, TGF-β did significantly increase the secretion of collagens on protein level (Figure 3C).

### 2.4. TNF-α Treatment Increased Cytokine Expression and Secretion in Cardiac Fibroblasts

**Figure 4:**
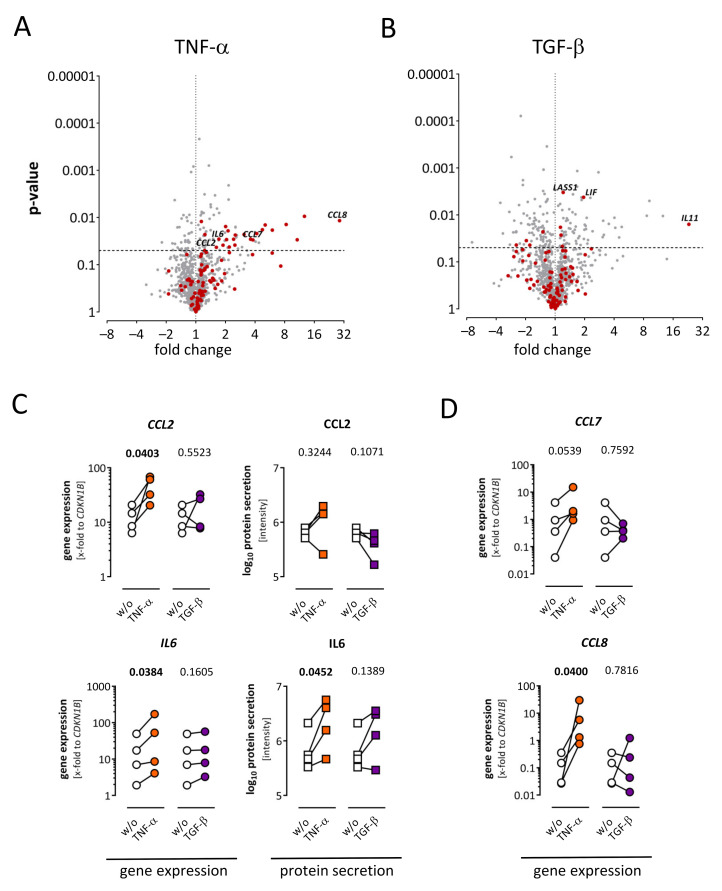
Regulation of cytokines in the secretome and corresponding transcriptome of human cardiac fibroblasts after TNF-α or TGF-β treatment. (**A**) Fold change and *p*-value are displayed in the volcano plot to visualize significantly regulated genes after TNF-α treatment. Genes assigned as cytokines are highlighted in red. Out of 110 significantly regulated genes (*p* < 0.05), 23 upregulated cytokines were detected. (**B**) Fold change and *p*-value are displayed in the volcano plot to show significantly regulated genes after TGF-β treatment. Genes assigned as cytokines are highlighted in red. Out of 174 overall significantly regulated genes (*p* < 0.05), six up- and four downregulated cytokines were detected. (**C**) After TNF-α (orange) or TGF-β (purple) stimulation, gene expression was assessed via TaqMan analysis (dots) and protein expression in the secretome (squares) were analyzed using mass spectrometry. Supporting Affymetrix results, gene expression of *CCL2* and *IL6* was significantly increased in the TaqMan analysis after TNF-α but not TGF-β treatment. On protein level, IL6 but not CCL2 was also increased. (**D**) Gene expression analysis via TaqMan analysis replicating Affymetrix results. After TNF-α (orange) and TGF-β (purple) stimulation, *CCL8* was increased in both gene expression analyses, while *CCL7* was significantly changed in the Affymetrix analysis (**A**). In the TaqMan analysis, *CCL7* was elevated in each sample but did not reach significance. Both genes were not detected by mass spectrometry. For statistical comparisons, either ratio paired *t*-test (gene expression data) or paired *t*-test (log_10_ protein data) was used as appropriate. Statistically significant results (*p* < 0.05) are highlighted in bold. ns, no significant differences; w/o, untreated controls.

The cytokine secretion of human cardiac fibroblasts after TNF-α or TGF-β treatment was further investigated on gene expression and protein level. As shown in Figure 4A,B, 1176 genes corresponding to secreted proteins were detected in the Affymetrix analysis and 96 of them were assigned as cytokines (red). After TNF-α stimulation, 110 genes were significantly regulated (73 upregulated and 37 downregulated). Of those 110 regulated genes, 23 were cytokines and all of them were upregulated (Figure 4A). After TGF-β stimulation, 174 genes were significantly regulated (80 upregulated and 94 downregulated). In contrast to the TNF-α stimulation, only 10 significantly regulated cytokines were detected, whereof six cytokines were upregulated and four cytokines were downregulated (Figure 4B).

For the genes *CCL2* and *IL6*, the results of Affymetrix analysis was verified by TaqMan gene expression analysis (Figure 4C). On protein level, IL6 was also upregulated after TNF-α treatment, while CCL2 was not regulated. Increased gene expression of *CCL8* was also confirmed by TaqMan analysis. In the case of *CCL7*, the gene expression was increased in Affymetrix results, while TaqMan analysis revealed increased expression in each of the four samples after TNF-α stimulation, but this did not pass significance level (Figure 4D). Both corresponding proteins were not detected using mass spectrometry.

### 2.5. Cardiac Fibroblasts Increased Collagen Secretion upon TGF-β Treatment

**Figure 5:**
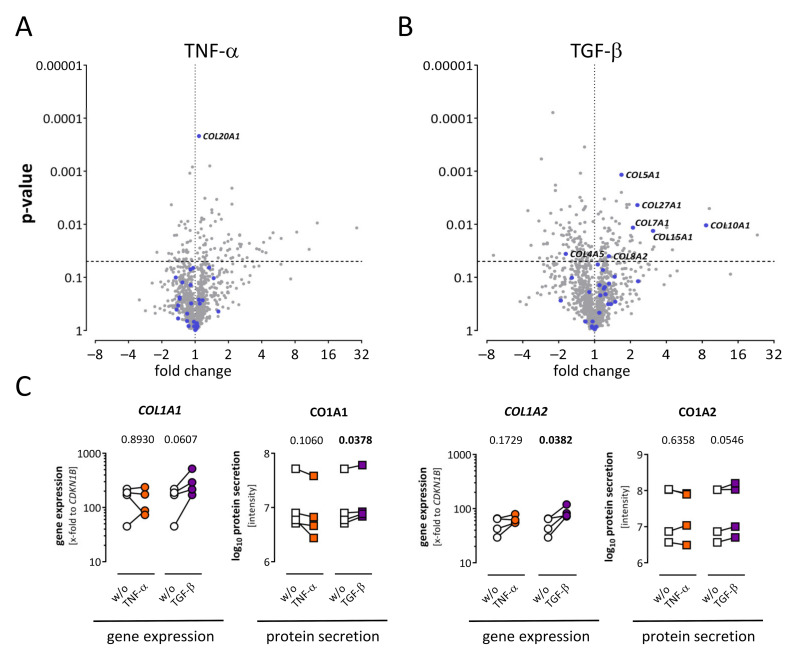
Regulation of collagens in the secretome and corresponding transcriptome of human cardiac fibroblasts. (**A**) Fold change and *p*-value are displayed in the volcano plot to visualize significantly regulated genes (*p* < 0.05) after TNF-α treatment. Genes assigned as collagens are highlighted in blue. One upregulated collagen was detected (*COL20A1*). (**B**) Fold change and *p*-value are displayed in the volcano plot to visualize significantly regulated genes (*p* < 0.05) after TGF-β treatment. Genes assigned as collagens are highlighted in blue. Six collagens were significantly upregulated and one collagen was downregulated. (**C**) After TNF-α (orange) or TGF-β (purple) stimulation, gene expression of selected genes was analyzed using TaqMan (dots), and secreted proteins were analyzed via mass spectrometry (squares). For statistical comparisons, either ratio paired *t*-test (gene expression data) or paired *t*-test (log_10_ protein data) was used as appropriate. Statistically significant results (*p* < 0.05) are highlighted in bold. ns, not significant; w/o, untreated control.

To investigate the role of TNF-α and TGF-β in cardiac fibrosis, collagen expression and secretion after stimulation was assessed in more detail. Only one out of 30 collagens was significantly regulated after TNF-α stimulation (*COL20A1*), shown in the volcano plot in Figure 5A. In contrast, after TGF-β stimulation, six collagens were significantly upregulated (Figure 5B). For five Affymetrix probe sets representing *COL1A1*, one was significantly regulated after TGF-β treatment (listed in Supplemental Table S3). Gene expression of *COL1A1* and *COL1A2* was additionally determined via TaqMan analysis (Figure 5C). Here, *COL1A1* expression was increased after TGF-β stimulation in each of the four patients without passing the significance level. The regulation of CO1A1 was also present on the protein level. In contrast, *COL1A2* was significantly upregulated in TaqMan analysis, while only one out of three Affymetrix probe sets representing COL1A2 was significantly upregulated (listed in Supplemental Table S3).

### 2.6. Matrix Metalloproteinases (MMPs) and Tissue Inhibitors of MMPs (TIMPs) Were Rarely Affected by Cytokine Treatment

**Figure 6:**
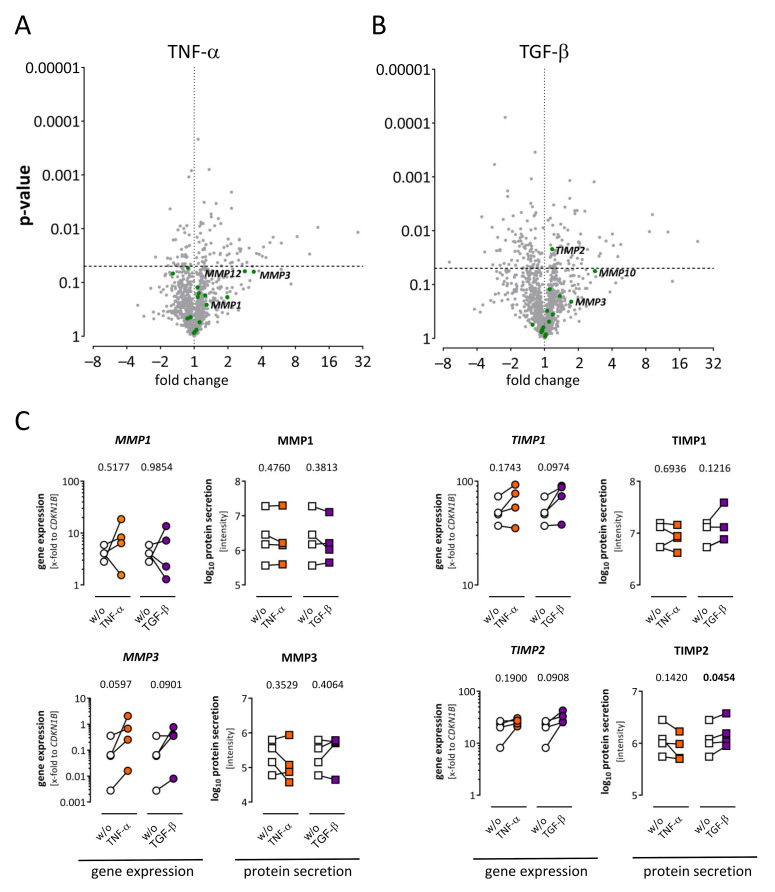
Alteration of matrix metalloproteinases (MMPs) and their endogenous inhibitors TIMPs in the secretome and corresponding transcriptome of human cardiac fibroblasts. (**A**) Fold change and *p*-value are displayed in the volcano plot to visualize significantly regulated genes after TNF-α treatment. Genes assigned as MMPs or TIMPs are highlighted in green. Significantly regulated MMPs or TIMPs were not detected, although *MMP3* and *MMP12* exhibited a high fold change. (**B**) Fold change and *p*-value are displayed in the volcano plot to visualize significantly regulated genes after TGF-β treatment. Genes assigned as MMPs or TIMPs are highlighted in green. A significant regulation (*p* < 0.05) was only observed for *TIMP2. (***C**) Alterations of selected molecules after TNF-α (orange) or TGF-β (purple) treatment on gene expression and protein level are shown. For gene expression TaqMan analysis results (dots) and for proteins, mass spectrometry-derived intensities (squares) are displayed. After TGF-β treatment, *TIMP2* was increased in each sample but did not reach significance on gene expression level and was significantly increased in the secretome. For statistical comparisons, either ratio paired *t*-test (gene expression data) or paired *t*-test (log_10_ protein data) was used as appropriate. Statistically significant results (*p* < 0.05) are highlighted in bold. ns, not significant; w/o, untreated controls.

MMPs and TIMPs are the major regulators of ECM degradation. Therefore, the quantification of differences depending on cytokine treatment was of particular interest. MMPs or TIMPs (green dots) were not significantly regulated after TNF-α treatment, as depicted in the volcano plot in Figure 6A. However, *MMP3* and *MMP12* exhibited a highly positive fold change. After TGF-β stimulation, *TIMP2* was significantly increased (Figure 6B). This result was partially confirmed by TaqMan gene expression analysis, where *TIMP2* was upregulated in each sample but did not pass the significance level (Figure 6C). On protein level, TIMP2 was significantly increased after TGF-β treatment. For other MMPs and TIMPs, there was no consistent regulation on gene expression, and on the protein level was not observed upon TNF-α nor upon TGF-ß treatment.

## 3. Discussion

TNF-α, as well as TGF-β-signaling, contribute to the pathophysiology of DCM. Since cardiac fibroblasts play a major role in the development of myocardial fibrosis, the effect of cytokine stimulation on cultured human cardiac fibroblasts derived from DCM patients was investigated in this study. Therefore, the secretome and the corresponding gene expression of human cardiac fibroblasts was investigated at a quiescent stage as well as under stimulation with TNF-α or TGF-β. The key findings of the current study are: (1) 58% of secreted proteins of a quiescent fibroblast were annotated to the ECM fraction, (2) cytokines are a substantial part of the fibroblast’s secretome on protein and gene expression level, (3) TNF-α stimulation of human cardiac fibroblasts preferentially induced gene expression of cytokines while TGF-β stimulation of human cardiac fibroblasts mainly induced collagen secretion. All conclusions are based upon findings with cultured primary fibroblasts.

In the present study, 112 different proteins were detected in the secretome of human cardiac fibroblasts by label-free mass spectrometry. Furthermore, the expression of almost 1200 genes coding for secreted proteins was detected in the Affymetrix analysis. All detected proteins revealed a high gene expression level. Since the most abundant proteins were also the highest expressed genes, a significant correlation between the amount of secreted proteins and corresponding gene expression was detected (Supplemental Figure S1). Nevertheless, more genes coding for secreted proteins than secreted proteins were observed, indicating a higher sensitivity for Affymetrix than for mass spectrometric analyses.

Out of 112 secreted proteins detected by mass spectrometry, 50 were annotated as ECM proteins, which were responsible for just over half of the protein amount (58%) of the whole secretome (Figure 2). In contrast to the typical definition of a fibroblast as a cell producing connective tissue [13], our findings are consistent with the more recently described roles of fibroblasts; as convertible cells with multiple and diverse functions besides ECM maintenance [1]. Out of 50 ECM proteins, 12 scaffold proteins—collagens and fibronectin—were detected. These scaffold proteins are responsible for almost half of the protein amount of the ECM fraction (48%). Myocardial tissue mainly consists of collagen type I (80%) and collagen type III (20%) [14,15]. In line with this, here, collagen type I was mainly detected in the secretome as well as in the corresponding transcriptome (Figure 2B). In contrast to previous studies [15,16,17], collagen type VI was highly abundant in the secretome as well as on gene expression level. This is consistent with gene expression data from the recently published single-cell RNA-sequencing dataset “heart cell atlas”, in which *COL6A1*, *COL6A2*, and *COL6A3* are also highly expressed in the fibroblast fraction [18].

Interestingly, cytokines, such as IL8 or IL6, were responsible for 5% of the total secretome amount, even at a quiescent stage (Figure 2). Our group showed previously that human cardiac fibroblasts could act as sentinel cells regulating inflammatory processes in reaction to mechanical stress [12,19]. Here, we could show that cardiac fibroblasts express 96 genes, annotated as cytokines, even at a quiescent stage, and eight proteins annotated as cytokines were detected in the secretome of quiescent fibroblasts (Figure 2).

MMPs and TIMPs were responsible for 11% of the protein amount of all secreted proteins. Mainly TIMP1 was detected, in line with gene expression data from the “heart cell atlas” [18]. Furthermore, MMP2 exhibited a higher protein amount than MMP1, but both were detected at high levels in the secretome as well as in the corresponding transcriptome. Furthermore, the “heart cell atlas” showed fibroblasts as the main source of *MMP2* in healthy cardiac tissue [18].

### 3.1. TNF-α Treatment Is Leading to Pro-Inflammatory Cytokine Expression but Not to Direct Fibrotic Effects

Stimulation of human cardiac fibroblast with TNF-α leads to a cytoplasm-to-nucleus translocation of the transcription factor NF-κB as shown in Figure 3A, indicating induction of gene expression. The induction of NF-κB-responsive, pro-inflammatory genes upon TNF-α stimulation was shown before in murine cardiac fibroblasts [20]. Several studies highlight that cardiac fibroblasts contribute to the inflammatory phase in many diseases [19,21,22]. Under stimulation with lipopolysaccharides or cytokines, cardiac fibroblasts produce pro-inflammatory cytokines and therefore contribute to leukocyte recruitment [22,23,24]. Upon stimulation with TNF-α, the overall gene expression of cytokines was increased (Figure 3B), while on protein level, only selected cytokines such as IL6 were upregulated. In detail, the gene expression of 23 cytokines, such as *CCL5*, *CCL8,* and *CXCL10,* significantly increased with a >3-fold change (Figure 4A). Furthermore, no downregulated cytokines were detected after TNF-α stimulation. Although MMP levels are elevated in a variety of inflammatory stages in cardiac diseases [25,26,27], fibroblasts may not be their primary source. While MMP1, MMP2, and MMP3 were detected as proteins in the secretome and also on gene expression level in quiescent fibroblasts (Figure 2), an increase after TNF-α stimulation was not observed in this study (Figure 3 and Figure 6). Since MMPs are secreted as inactive pro-forms, an increase in MMP activity upon TNF-α treatment, as shown previously [28], might not be caused by alterations of gene expression or protein secretion. Moreover, TNF-α stimulation was not leading to an increased collagen expression or secretion (Figure 3 and Figure 5), indicating that TNF-α is leading to a pro-inflammatory, but not to a direct, fibrotic remodeling.

### 3.2. TGF-β Treatment Is Causing Pro-Fibrotic Effects

In general, the role of TGF-β mediating pro-fibrotic effects in cardiac fibroblasts is already described [1,11], but untargeted approaches, as well as the comparison of gene expression and protein secretion, are lacking. The activation of the SMAD2/3 transcription factor is known to play an important role in TGF-β signaling [29]. Its translocation from cytoplasm to nucleus (Figure 3A) upon TGF-β stimulation has been shown in murine cardiac fibroblasts previously and is contributing to fibrosis [30,31]. In our previous studies, we already confirmed the transdifferentiation of fibroblasts to myofibroblasts followed by TGF-β stimulation [12,19]. Focusing on the secretome, this study shows that the overall secretion of collagens was increased in human cardiac fibroblasts after TGF-β stimulation, while the response on gene expression of corresponding genes was not significant (Figure 3C). On the single protein level also, CO1A1, CO4A2, CO5A1, and CO5A2 increased significantly in the secretome (Figure 5). It can be speculated that the highest expression of those genes is already reached a few hours after the beginning of TGF-β stimulation. However, gene expression of other collagens, which were not detectable in the secretome by mass spectrometry, was increased. In contrast to TNF-α, the overall expression and secretion of cytokines were not affected by TGF-β treatment. However, *IL11* gene expression was highly increased after TGF-β treatment. IL11 previously showed a strong pro-fibrotic effect downstream of TGF-β signaling in human cardiac fibroblasts and an angiotensin-II mouse model [32], which is in line with our findings. Although TGF-β has been associated with increased secretion of TIMPs earlier [1,33], increased expression and secretion was observed only for TIMP2 in this study. Taken together, our data support the pro-fibrotic role of TGF-β, which is mainly driven by an increased secretion of collagens.

### 3.3. Limitations

Despite using low passages of primary cardiac fibroblasts, this experimental approach cannot exclude confounding factors induced by cultivation on plastic cell culture dishes. Furthermore, since only fibroblasts derived from DCM patients were used for the analyses realized in this study, it is not clear whether fibroblasts derived from healthy myocardium might respond similarly. However, this approach was in line with the aim of the study investigating pathomechanisms of the remodeling process in DCM. Furthermore, since the number of human samples is low, statistical analysis has limited power.

## 4. Materials and Methods

### 4.1. Study Population and Cell Culture

Patients with dilated cardiomyopathy (EF < 45%) were included in this study. Before obtaining endomyocardial biopsies, coronary artery disease was excluded and ejection fraction was evaluated by echocardiography. The patient’s characteristics are summarized in Supplemental Table S6. All patients provided their written informed consent for invasive diagnostic procedures. The research protocol was approved by the local institutional review committee of the Charité Berlin, Benjamin-Franklin Campus (no: 225-07, approved 2008).

Human cardiac fibroblasts were obtained by outgrowth from endomyocardial biopsies as described previously [12,19]. Cells were cultured in Iscove’s basal medium (Biochrom AG, Berlin, Germany) containing 10% human serum, 10% fetal calf serum (Biochrom AG, Berlin, Germany), 100 U/mL penicillin, and 100 µg/mL streptomycin (Sigma-Aldrich, Taufkirchen, Germany). All experiments with those primary cells were carried out between passages 2 and 3 [12,34].

### 4.2. Cytokine Stimulation

Human cardiac fibroblasts were seeded into 6-well plates and grown to confluence in supplemented Iscove’s basal medium (Biochrom AG, Berlin, Germany) as described above. Before stimulation, cells were serum-starved in a reduced medium, Iscove’s basal medium with 0.5% fetal calf serum (Biochrom AG, Berlin, Germany), 100 U/mL penicillin, and 100 µg/mL streptomycin (Sigma-Aldrich, Taufkirchen, Germany) overnight. Afterwards, cells were stimulated either with 10 ng/mL recombinant human TNF-α (Peprotech, Hamburg, Germany) or 5 ng/mL recombinant human TGF-β (Peprotech, Hamburg, Germany), diluted in serum-reduced Iscove medium. Cells treated equally without the addition of TNF-α or TGF-β and incubated for the same time periods were used as controls. Depending on the experiment, cells were stimulated from 6 to 72 h.

### 4.3. Immunofluorescence Staining

Human cardiac fibroblasts were seeded in chamber slides and grown to confluence with supplemented Iscove’s basal medium (Biochrom AG, Berlin, Germany) as described above. Cells were stimulated either with TNF-α or TGF-β for 6 h and fixed with 4% paraformaldehyde for subsequent fluorescence staining. Antibodies directed against NF-κB p65 ((D14E12) XP^®^ rabbit mAb #8242) or Smad2/3 ((D7G7) XP^®^ rabbit mAb #8685) (Cell Signaling, Frankfurt, Germany) were used. For visualization, Alexa Fluor 594 conjugated goat anti-rabbit IgG (H+L) was applied as a secondary antibody (R37117, Thermo Fisher Scientific, Schwerte, Germany). To visualize the cell shape, Alexa Fluor^®^ 488 Phalloidin (A12379, Thermo Fisher Scientific) was used to stain F-Actin and Hoechst 33,342 (B2261, Sigma-Aldrich, Taufkirchen, Germany) was used to stain nuclei. Documentation of the staining was done with an Axiovert M200 microscope equipped with an ApoTome modul (Zeiss, Jena, Germany).

### 4.4. Mass Spectrometric Analysis of the Extracellular Proteome

To analyze the extracellular proteome, human cardiac fibroblasts from four different patients were stimulated as described above, and the cell culture supernatant was collected after 72 h. Proteins in the supernatant were precipitated with 15% trichloro acetic acid and reconstituted in 30 µL of 8 M urea and 2 M thiourea. The protein concentration was determined by Bradford protein assay (Biorad, Munich, Germany). Four µg of extracellular proteins per sample were subjected to reduction and alkylation before being digested with trypsin (enzyme to protein ratio 1:50) overnight at 37 °C. After stopping the proteolysis by adding acetic acid in a final concentration of 1%, peptide extracts were purified on C18 reverse phase material (µZip Tip, Merck Millipore, Darmstadt, Germany). The purified samples were analyzed by nano-LC-ESI tandem mass spectrometry on an Easy nLC II-System (Proxeon, Odense, Denmark) online coupled to a LTQ Orbitrap Velos mass spectrometer (Thermo Electron, Bremen, Germany) operated in data-dependent mode. Data alignment, feature extraction, and peptide identification using SEQUEST-algorithm were carried out by Rosetta Elucidator Software (Ceiba Solution Inc., Boston, MA, USA). For identification, a combined Uniprot/SwissProt database (v. 2014_10) for bovine and human sequences was used. Peptides were annotated at a false discovery rate < 1% and a protein teller score > 0.9 was applied. Shared peptides for human and bovine proteins were excluded and quantitative analyses were carried out for human protein-specific sequences only. Further details are given in Supplemental Material Table S7.

Gene Data Analyst 8.2. (Gene Data, Basel, Switzerland) was used for relative quantitative analysis. Protein intensities were log_10_ transformed and median normalized before application of paired *t*-test for the identification of differences in protein levels. The threshold for significance was set to *p* < 0.05. Protein portions within samples were calculated from intensity-based absolute quantification (iBAQ) values [35]. iBAQ values were calculated by dividing the intensities by the number of theoretical tryptic peptides in the protein.

### 4.5. RNA Isolation

Human cardiac fibroblasts derived from four different patients were cultured and stimulated as described above. Each experiment was carried out in 4–6 replicates. After 24 h of treatment with TNF-α or TGF-β, cells were lysed immediately in RLT-buffer (Qiagen, Hilden, Germany) containing 1% mercaptoethanol. Total RNA was isolated using RNeasy Mini kit (Qiagen, Hilden, Germany) according to the manufacturer’s instructions using RNeasy columns. RNase free DNase I (Qiagen, Hilden, Germany) was directly used on the column to digest genomic DNA. The yield of eluted total RNA was determined by measuring the UV absorbance at 260 nm on the Nanodrop 2000c spectrophotometer (Thermo Fisher Scientific, Schwerte, Germany) and stored at –80 °C until further analyses.

### 4.6. Quantitative Gene Expression Analysis Using TaqMan

For quantitative real-time TaqMan PCR, 250 ng total RNA was reversely transcribed into cDNA using a high capacity kit (Thermo Fisher Scientific, Schwerte, Germany) and subsequently diluted to a final contraction of 1.25 ng/µL. Subsequently, 5 µL gene expression Mastermix (Thermo Fisher Scientific, Schwerte, Germany) and 0.5 µL of TaqMan gene expression assay purchased from Thermo Fisher Scientific (Schwerte, Germany) were used in a final volume of 10 µL including 1 µL of cDNA template (performed in duplicates). As an internal control, for each cDNA, the gene expression of the housekeeping gene *CDKN1B* was quantified and verified that it was not affected by treatments. Gene expression of target genes was normalized to the determining gene expression level of *CDKN1B* using the formula 2^−ΔCt^. Expression data were displayed as mean values for untreated and treated samples and visualized as spaghetti plots for each patient separately using GraphPad Prism 6.05 (GraphPad Software, La Jolla, CA, USA). Statistical comparison of two groups was performed using the ratio paired *t*-test with *p*-values < 0.05 considered as statistically significant.

### 4.7. Quantitative Gene Expression Analysis Using Affymetrix Arrays

The GeneChip^®^ Human Genome U133 Plus 2.0 array (Affymetrix, Santa Clara, CA, USA) comprises over 47,000 transcripts. RNA integrity was checked by analyzing on the 2100 Bioanalyzer (Agilent Technologies, Santa Clara, CA, USA). For the first cycle of cDNA synthesis, 300 ng of total RNA was used. During the following in vitro transcription reaction, cRNA was obtained and used as a template for the second cycle of cDNA synthesis. The second cycle random-primed single-strand DNA synthesis resulted in a product containing incorporated deoxyuridine at predefined ratios relative to thymidine (Ambion^®^ WT Expression Kit, Ambion, Austin, TX, USA). Subsequently, 5.5 µg of this generated single-strand DNA was fragmented with a combination of uracil DNA glucosylase (UDG) and apurinic/apyrimidinic endonuclease 1 (APE1) and then labeled using the WT terminal labeling kit (Affymetrix, Santa Clara, CA, USA). The biotinylated DNA was added to a hybridization cocktail for hybridization to the Human Genome U133 Plus 2.0 array in the Affymetrix hybridization oven 640 at 45 °C and 60 rpm for 16 h. Staining and washing, processed in the Fluidics Station 450, followed by scanning on the GeneChip Scanner 3000 G7 System, were carried out as recommended by Affymetrix (Affymetrix, Santa Clara, CA, USA). The CEL files were imported to the Partek Genomics Suite 6.6 software. The data processing steps involved the background correction on the PM values, the quantile probe normalization across all arrays of the experiment as well as the log_2_ transformation. A mean value of 5 was set to be the threshold for a detectable expression in the investigated samples. Genes were excluded from subsequent analysis if none or only one out of 12 samples reached the threshold level. To visualize significantly regulated genes as volcano plots, fold changes were plotted on a log_2_-*x*-axis and *p*-values on a log_10_-*y*-axis using GraphPad Prism 6.05 (GraphPad Software, La Jolla, CA, USA).

### 4.8. Identification of Genes Coding for Secreted Proteins Using Uniprot

For further analyses, genes coding for secreted proteins were extracted out of the Affymetrix data. For this purpose, Uniprot accession numbers of all secreted proteins were converted into Affymetrix IDs using the manufacturer identifier R object ”hgu133plus2.db” (Version 3.13.0, July 2021) together with the R package AnnotationDbi (Version 1.54.1, June 2021). If genes were linked to more than one Affymetrix ID, the highest expressed ID in the control samples was used. Out of 2091 secreted proteins annotated by Uniprots subcellular locations database, 1815 unique genes were assigned, whereof 1176 reached the threshold level. To visualize differentially expressed genes by treatment, log_2_ fold change of gene expression and *p*-values were plotted as a volcano-plot using GraphPad Prism 6.05 (GraphPad Software, La Jolla, CA, USA). Statistical comparison of treated vs. untreated samples was performed using the paired *t*-test with *p*-values < 0.05 considered as statistically significant.

### 4.9. Assigning Proteins and Genes to Their Subcategory

For the analyses of the secretome and corresponding transcriptome all genes were assigned to the categories “secreted”, “membrane”, “endoplasmic riticulum”, “extracellular matrix”, and “cytokines” with the help of the Uniprot database “uniprot keyword”. Here, only reviewed proteins out of the Swiss-Prot database were used for the annotations. For the categories “collagens” and “MMPs/TIMPs” all proteins were assigned according to their names.

## 5. Conclusions

Overall our data suggest that human cardiac fibroblasts undergo dynamic adaptions in response to extracellular stimuli. Alterations in secretome composition as well as in underlying gene expression indicate changes in the functional role of cardiac fibroblasts during pathophysiological processes of DCM. This gives another hint that fibroblasts are contributing both to inflammatory as well as to pro-fibrotic processes in the remodeling of the myocardium during DCM.

## Data Availability

The data presented in this study are available in the Supplementary Material.

## Supplementary Materials

The following are available online at https://www.mdpi.com/article/10.3390/ijms222212262/s1. Figure S1: Correlation of protein abundance and gene expression. Abundance of secreted proteins was measured by quantified mass spectrometry, while gene expression of corresponding genes was analyzed via Affymetrix. Cytokines are colored in red, Collagens in blue and MMPs/TIMPs in green. Log_10_ (protein) or log_2_ (gene expression) data is plotted on linear axis. Correlation was calculated using Pearson test. Table S1: Composition of the extracellular proteome of human cardiac fibroblasts. Fractions were calculated according to the protein intensity in a mass spectrometric analysis of 500 ng protein of the culture supernatant obtained by precipitation with TCA. Proteins were categorized according to the localization annotation in Uniprot (2015). Data are summarized in Figure 2A. Table S2: Affymetrix data from human cardiac fibroblasts after stimulation for all expressed cytokines (mean values ≥ 5). Human cardiac fibroblasts derived from four patients were incubated with or without 10 ng/mL TNF-α or 5 ng/mL TGF-β for 24 h followed by RNA extraction and subsequent gene expression analysis using Affymetrix arrays. Raw data are listed in alphabetic order. Statistical analysis was performed using paired *t* test. Table S3: Affymetrix data from human cardiac fibroblasts after stimulation for all expressed collagens. Human cardiac fibroblasts derived from four patients were incubated with or without 10 ng/mL TNF-α or 5 ng/mL TGF-β for 24 h followed by RNA extraction and subsequent gene expression analysis using Affymetrix arrays. Raw data are listed in alphabetic order. Statistical analysis was performed using paired *t* test. Table S4: Affymetrix data from human cardiac fibroblasts after stimulation for all expressed MMPs and TIMPs. Human cardiac fibroblasts derived from four patients were incubated with or without 10 ng/mL TNF-α or 5 ng/mL TGF-β for 24 h followed by RNA extraction and subsequent gene expression analysis using Affymetrix arrays. Raw data are listed in alphabetic order. Statistical analysis was performed using paired *t* test. Table S5: Impact of TNF-α and TGF-β on the extracellular proteome of human cardiac fibroblasts in comparison to untreated cells. Human cardiac fibroblasts derived from four patients were incubated with or without 10 ng/mL TNF-α or 5 ng/mL TGF-β for 72 h followed by protein extraction from culture supernatants by TCA precipitation and analysis of 500 ng tryptic peptides by tandem mass spectrometry. Shown are fold changes in comparison to proteins derived from untreated control samples as well as the group means and the p-values. Statistical analysis was performed using paired *t* test. Table S6: Cardiac fibroblasts derived from patients with the following characteristics. Table S7: Description of experiment settings for LC-ESI-MS analysis. Parameters for liquid chromatography and search parameter to identify the proteins are listed.
