# Supplementary material for: Cytokine-Mediated Alterations of Human Cardiac Fibroblast’s Secretome
Source: Int J Mol Sci. 2021 Nov 12;22(22):12262. doi: 10.3390/ijms222212262 (PMC8617966; doi:10.3390/ijms222212262)
Supplement: Supplementary file 1 [file ijms-22-12262-s001.zip › ijms-1421956-supplementary.pdf]

Supplementary Material

Cytokine-Mediated Alterations of Human Cardiac Fibroblast's Secretome

Hanna Bräuninger [1,2*], Tilo Thottakara [1,2*], Jacob Schön [3*], Svenja Voss [1,2], Vishnu Dhople [3,4], Svenja Warnke [1], Katharina Scherschel [1,5,6], Benedikt N. Schrage [1,2], Paulus Kirchhof [1,2,7], Stefan Blankenberg [1,2], Uwe Völker [3,4], Dirk Westermann [1,2], Elke Hammer [3,4*], Diana Lindner [1,2*]✉

[1] Clinic for Cardiology, University Heart & Vascular Centre, University Hospital Hamburg-Eppendorf, Martinistrasse 52, 20246 Hamburg, Germany
[2] DZHK (German Centre for Cardiovascular Research), Partner Site Hamburg/Kiel/Lübeck, 20246 Hamburg, Germany
[3] Interfaculty Institute for Genetics and Functional Genomics, Department of Functional Genomics, University Medicine Greifswald, Friedrich-Ludwig-Jahn-Str. 15a, D-17475 Greifswald, Germany
[4] DZHK (German Centre for Cardiovascular Research), Partner Site Greifswald, Greifswald, Germany
[5] Division of Cardiology (cNEP), EVK Düsseldorf, Germany
[6] Institute of Neural and Sensory Physiology, Medical Faculty, Heinrich Heine University Düsseldorf, Germany
[7] Institute of Cardiovascular Sciences, University of Birmingham, UK
* Correspondence: e-mail@e-mail.com

1. Expanded Results

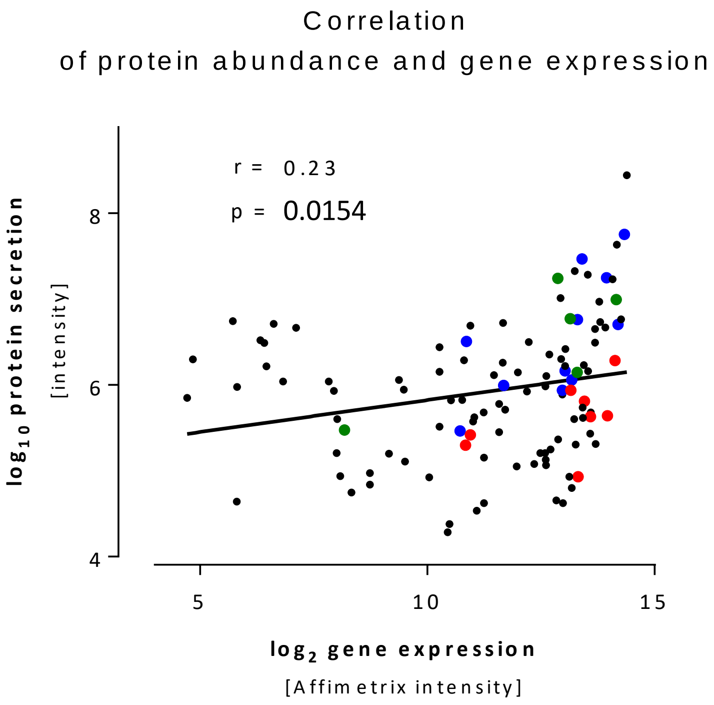

Supplemental Figure S1: Correlation of protein abundance and gene expression. Abundance of secreted proteins was measured by quantified mass spectrometry, while gene expression of corresponding genes was analyzed via Affymetrix. Cytokines are colored in red, Collagens in blue and MMPs/TIMPs in green. Log_{10} (protein) or log_2 (gene expression) data is plotted on linear axis. Correlation was calculated using Pearson test.

Supplemental Table S1: Composition of the extracellular proteome of human cardiac fibroblasts. Fractions were calculated according to the protein intensity in a mass spectrometric analysis of 500 ng protein of the culture supernatant obtained by precipitation with TCA. Proteins were categorized according to the localization annotation in Uniprot (2015). Data are summarized in Figure 2A.

No	Protein symbol	Swiss Prot number	Protein name	intensity, log10				iBAQ value	fraction of total [%]
				untr. #1	untr. #2	untr. #3	untr. #4		
secreted ECM proteins									
1	FINC	P02751	Fibronectin	8.52	8.08	8.11	8.72	2759295	12.25
2	POSTN	Q15063	Periostin	7.75	7.16	7.44	7.86	976851	4.34
3	TIMP1	P01033	Metalloproteinase inhibitor 1	7.19	6.73	6.72	7.12	894021	3.97
4	CO1A2	P08123	Collagen alpha-2(I) chain (Alpha-2 type I collagen)	8.03	6.57	6.87	8.04	752751	3.34
5	LEG1	P09382	Galectin-1	6.61	6.93	6.88	6.49	644534	2.86
6	BGH3	Q15582	Transforming growth factor-beta-induced protein ig-h3	7.35	7.52	7.03	7.27	541676	2.40
7	CO6A1	P12109	Collagen alpha-1(VI) chain	7.63	7.33	6.88	7.66	540537	2.40
8	MMP2	P08253	72 kDa type IV collagenase	6.78	7.11	6.70	7.66	481833	2.14
9	MMP1	P03956	Interstitial collagenase	6.17	7.28	6.45	5.56	246210	1.09
10	CO1A1	P02452	Collagen alpha-1(I) chain (Alpha-1 type I collagen)	6.90	6.71	6.77	7.72	227102	1.01
11	FBLN3	Q12805	EGF-containing fibulin-like extracellular matrix protein 1	6.60	6.37	6.28	7.02	178509	0.79
12	TSP1	P07996	Thrombospondin-1	6.88	6.51	7.07	7.17	138718	0.62
13	TIMP2	P16035	Metalloproteinase inhibitor 2	6.08	6.00	5.74	6.45	126661	0.56
14	LG3BP	Q08380	Galectin-3-binding protein	6.52	5.91	6.11	6.75	98287	0.44
15	CO3A1	P02461	Collagen alpha-1(III) chain	6.89	5.98	6.29	6.98	71046	0.32
16	CO4A2	P08572	Collagen alpha-2(IV) chain [Cleaved into: Canstatin]	6.60	6.00	6.41	7.19	67016	0.30
17	PGS1	P21810	Biglycan	5.66	4.85	5.06	6.66	65010	0.29
18	CO6A2	P12110	Collagen alpha-2(VI) chain	6.45	6.26	6.22	6.81	60276	0.27
19	HPLN1	P10915	Hyaluronan and proteoglycan link protein 1	5.67	5.76	6.02	6.48	55153	0.24
20	LTBP2	Q14767	Latent-transforming growth factor beta-binding protein 2	6.03	6.48	6.27	6.82	54199	0.24
21	LOXL2	Q9Y4K0	Lysyl oxidase homolog 2	6.29	6.04	6.06	6.14	39774	0.18
22	ECM1	Q16610	Extracellular matrix protein 1	6.14	5.67	6.02	6.22	34679	0.15

23	FBLN1	P23142	Fibulin-1	6.20	5.87	6.13	5.85	32299	0.14
24	PGBM	P98160	Basement membrane-specific heparan sulfate proteoglycan core protein	6.65	6.35	6.44	7.06	30737	0.14
25	LAMC1	P11047	Laminin subunit gamma-1	6.29	6.22	6.07	6.63	29324	0.13
26	FBN1	P35555	Fibrillin-1	6.79	6.27	6.41	6.86	28894	0.13
27	A1AT	P01009	Alpha-1-antitrypsin	5.37	6.02	6.07	5.56	28201	0.13
28	CALR	P27797	Calreticulin	5.64	5.92	5.91	5.49	24894	0.11
29	PXDN	Q92626	Peroxidasin homolog	6.38	6.26	6.16	6.36	24628	0.11
30	CCD80	Q76M96	Coiled-coil domain-containing protein 80	6.16	5.47	5.92	6.10	18185	0.08
31	MFAP5	Q13361	Microfibrillar-associated protein 5	4.73	5.09	4.80	5.61	17881	0.08
32	CTGF	P29279	Connective tissue growth factor	5.88	5.20	5.63	5.75	17065	0.08
33	CO4A1	P02462	Collagen alpha-1(IV) chain [Cleaved into: Arresten]	6.12	5.91	5.98	6.17	14634	0.06
34	MMP3	P08254	Stromelysin-1	5.16	5.80	5.55	4.77	11911	0.05
35	NID2	Q14112	Nidogen-2	6.26	4.75	5.12	5.82	11463	0.05
36	CO5A2	P05997	Collagen alpha-2(V) chain	5.77	5.20	5.43	6.39	11344	0.05
37	CSPG2	P13611	Versican core protein	5.60	5.66	5.87	6.62	11185	0.05
38	CO5A1	P20908	Collagen alpha-1(V) chain	5.86	5.65	5.93	6.29	10846	0.05
39	LAMB1	P07942	Laminin subunit beta-1	5.79	5.87	5.52	6.22	9615	0.04
40	CTHR1	Q96CG8	Collagen triple helix repeat-containing protein 1	5.30	5.22	4.67	4.82	9200	0.04
41	CO6A3	P12111	Collagen alpha-3(VI) chain	5.76	5.80	5.84	6.59	8146	0.04
42	NID1	P14543	Nidogen-1	5.59	4.72	5.06	5.87	6663	0.03
43	LAMA4	Q16363	Laminin subunit alpha-4	5.60	5.81	5.56	6.09	6251	0.03
44	LTBP1	Q14766	Latent-transforming growth factor beta-binding protein 1	5.86	5.75	5.10	5.41	5600	0.02
45	TICN1	Q08629	Testican-1	5.04	4.68	4.81	5.39	5045	0.02
46	TENA	P24821	Tenascin	5.74	5.20	5.24	5.79	3569	0.02
47	PGS2	P07585	Decorin	5.03	3.53	3.55	4.73	2004	0.01
48	COCA1	Q99715	Collagen alpha-1(XII) chain	5.61	5.01	5.17	5.71	1859	0.01
49	BMP1	P13497	Bone morphogenetic protein 1	5.08	4.63	5.09	4.96	1647	0.01
50	AGRIN	O00468	Agrin	4.60	4.50	4.50	5.39	915	0.00

								**9438143**	**41.90**
**secreted cytokines**									
1	IL8	P10145	Interleukin-8	5.67	6.81	5.52	5.67	382957	2
2	CCL2	P13500	C-C motif chemokine 2	5.90	5.80	5.71	5.81	161170	1
3	CXCL6	P80162	C-X-C motif chemokine 6	5.69	5.90	5.26	5.40	85512	0
4	IL6	P05231	Interleukin-6	5.74	6.33	5.52	5.67	66706	0
5	GROA	P09341	Growth-regulated alpha protein	5.08	6.14	5.08	5.14	54727	0
6	GDF15	Q99988	Growth/differentiation factor 15	5.12	5.45	5.33	5.62	15376	0
7	GREM1	O60565	Gremlin-1	4.83	5.31	4.55	4.51	9450	0
8	G6PI	P06744	Glucose-6-phosphate isomerase	4.94	5.55	5.40	4.98	6005	0
								781903	3.16
**other secreted proteins**									
1	IBP7	Q16270	Insulin-like growth factor-binding protein 7	7.42	6.99	7.04	7.33	1132549	5.03
2	IGKC	P01834	Ig kappa chain C region	6.18	6.87	7.03	5.95	1022022	4.54
3	PAI1	P05121	Plasminogen activator inhibitor 1	7.39	7.34	7.12	7.24	955845	4.24
4	B2MG	P61769	Beta-2-microglobulin	6.60	6.56	6.24	7.09	672997	2.99
5	IGHG1	P01857	Ig gamma-1 chain C region	6.22	6.76	7.03	5.50	307695	1.37
6	CYTC	P01034	Cystatin-C	6.47	5.94	5.95	6.48	193044	0.86
7	APOA1	P02647	Apolipoprotein A-I	6.10	6.63	6.84	5.89	157265	0.70
8	GDN	P07093	Glia-derived nexin	6.00	6.06	6.19	6.94	147105	0.65
9	PTX3	P26022	Pentraxin-related protein PTX3	6.59	5.30	5.79	6.76	145649	0.65
10	TRFE	P02787	Serotransferrin	6.28	6.93	7.00	6.19	128221	0.57
11	CO3	P01024	Complement C3	6.40	6.86	6.89	7.37	96000	0.43
12	HPT	P00738	Haptoglobin	5.78	6.41	6.64	5.59	90307	0.40
13	IBP3	P17936	Insulin-like growth factor-binding protein 3	5.99	6.05	6.09	6.54	89175	0.40
14	VTNC	P04004	Vitronectin	6.30	5.83	6.38	6.18	78415	0.35
15	CFAH	P08603	Complement factor H	6.20	6.54	6.49	7.05	75011	0.33
16	ITIH2	P19823	Inter-alpha-trypsin inhibitor heavy chain H2	6.63	6.13	6.62	6.51	63750	0.28

17	IGHA1	P01876	Ig alpha-1 chain C region	5.35	6.10	6.34	4.96	62827	0.28
18	APOA2	P02652	Apolipoprotein A-II	5.01	5.75	5.66	4.96	60110	0.27
19	SAP	P07602	Prosaposin	6.00	6.37	6.20	6.22	50058	0.22
20	CALU	O43852	Calumenin	5.97	6.01	5.86	5.60	45298	0.20
21	PCOC1	Q15113	Procollagen C-endopeptidase enhancer 1	6.34	5.60	5.10	5.89	41760	0.19
22	A2MG	P01023	Alpha-2-macroglobulin	6.56	6.36	6.73	5.95	40930	0.18
23	STC2	O76061	Stanniocalcin-2	5.57	5.62	5.55	5.96	39492	0.18
24	C1R	P00736	Complement C1r subcomponent	6.18	5.16	5.37	6.58	36427	0.16
25	QSOX1	O00391	Sulfhydryl oxidase 1	6.29	6.16	6.03	6.45	36224	0.16
26	SRGN	P10124	Serglycin	5.64	4.64	4.92	5.40	34137	0.15
27	IGHG2	P01859	Ig gamma-2 chain C region	5.03	5.68	5.99	4.82	31347	0.14
28	NUCB1	Q02818	Nucleobindin-1	6.01	5.77	5.41	6.19	28425	0.13
29	B4GT1	P15291	Beta-1,4-galactosyltransferase 1	5.34	5.40	5.70	5.98	26621	0.12
30	LYOX	P28300	Protein-lysine 6-oxidase	5.88	5.30	5.43	5.62	24170	0.11
31	IBP6	P24592	Insulin-like growth factor-binding protein 6	5.95	4.95	4.43	5.09	23377	0.10
32	EDIL3	O43854	EGF-like repeat and discoidin I-like domain-containing protein 3	5.56	5.51	5.54	6.05	20067	0.09
33	FSTL1	Q12841	Follistatin-related protein 1	5.57	5.44	5.15	5.91	17401	0.08
34	THIO	P10599	Thioredoxin	4.88	4.93	5.21	4.23	12139	0.05
35	CATB	P07858	Cathepsin B	5.02	5.58	5.14	4.94	11077	0.05
36	DKK3	Q9UBP4	Dickkopf-related protein 3	5.44	4.64	4.64	5.45	9469	0.04
37	FAM3C	Q92520	Protein FAM3C	5.26	4.80	4.72	5.18	7499	0.03
38	ANT3	P01008	Antithrombin-III	5.09	4.81	5.44	5.31	5551	0.02
39	GAS6	Q14393	Growth arrest-specific protein 6	4.54	5.28	5.42	5.64	5243	0.02
40	INHBA	P08476	Inhibin beta A chain	4.81	5.13	4.74	5.50	4749	0.02
41	ADAM9	Q13443	Disintegrin and metalloproteinase domain-containing protein 9	5.36	5.34	4.88	5.45	4464	0.02
42	PAPP1	Q13219	Pappalysin-1	5.45	5.58	5.28	5.35	3974	0.02
43	CFAB	P00751	Complement factor B	5.06	4.94	4.84	5.55	3946	0.02
44	TFPI1	P10646	Tissue factor pathway inhibitor	4.77	4.32	4.76	4.64	3485	0.02

45	A4	P05067	Amyloid beta A4 protein	5.01	5.11	5.03	5.31	3290	0.01
46	ANXA1	P04083	Annexin A1	4.67	4.96	4.99	4.24	2995	0.01
47	IGHG3	P01860	Ig gamma-3 chain C region	4.02	4.93	4.87	3.61	2905	0.01
48	CATD	P07339	Cathepsin D	4.94	4.08	4.68	4.88	2801	0.01
49	PLTP	P55058	Phospholipid transfer protein	4.90	4.51	4.04	5.18	2753	0.01
50	GELS	P06396	Gelsolin	4.86	4.45	4.96	5.16	2400	0.01
51	NUCB2	P80303	Nucleobindin-2	4.58	4.33	4.00	4.83	1426	0.01
52	SFRP4	Q6FHJ7	Secreted frizzled-related protein 4	4.31	3.96	4.04	4.74	1410	0.01
53	FNDC1	Q4ZHG4	Fibronectin type III domain-containing protein 1	4.62	4.64	4.46	5.60	1282	0.01
54	ITGBL	O95965	Integrin beta-like protein 1	3.50	3.54	4.00	4.78	739	0.00
								6067321	26.93
			**membrane or ER proteins**						
1	LMBD2	Q68DH5	LMBR1 domain-containing protein 2	8.21	8.03	8.16	8.07	4753179	21.10
2	VIME	P08670	Vimentin	7.01	6.97	7.05	6.63	264912	1.18
3	ENOA	P06733	Alpha-enolase	6.05	6.68	6.42	5.90	83946	0.37
4	G3P	P04406	Glyceraldehyde-3-phosphate dehydrogenase	5.95	6.15	6.21	5.32	51646	0.23
5	PLOD2	O00469	Procollagen-lysine,2-oxoglutarate 5-dioxygenase 2	6.02	6.52	6.39	6.01	47867	0.21
6	PDIA1	P07237	Protein disulfide-isomerase	6.07	6.40	6.42	6.02	47080	0.21
7	SERPH	P50454	Serpin H1	5.53	5.86	5.81	5.73	22394	0.10
8	PPIB	P23284	Peptidyl-prolyl cis-trans isomerase B	5.47	5.54	5.48	5.24	18650	0.08
9	PDIA3	P30101	Protein disulfide-isomerase A3	5.39	5.81	5.81	5.39	13882	0.06
10	PLOD1	Q02809	Procollagen-lysine,2-oxoglutarate 5-dioxygenase 1	5.79	5.64	5.45	5.90	11305	0.05
11	CSTN1	O94985	Calsyntenin-1	5.55	5.43	5.46	5.91	10492	0.05
12	MOES	P26038	Moesin	5.30	5.62	5.76	5.32	8770	0.04
13	BASP1	P80723	Brain acid soluble protein 1	4.34	5.26	5.14	4.90	8746	0.04
14	CAB45	Q9BRK5	45 kDa calcium-binding protein	5.30	5.11	4.46	4.97	5940	0.03
15	PDIA6	Q15084	Protein disulfide-isomerase A6	4.76	5.51	4.99	4.69	5473	0.02
16	VINC	P18206	Vinculin	5.55	5.61	5.73	5.34	4551	0.02

17	CADH2	P19022	Cadherin-2	4.87	4.82	5.00	5.43	3740	0.02
18	LDHB	P07195	L-lactate dehydrogenase B chain	4.94	5.04	4.89	4.46	3615	0.02
19	GDIB	P50395	Rab GDP dissociation inhibitor beta	4.91	5.07	5.15	4.82	3490	0.02
20	COR1C	Q9ULV4	Coronin-1C	4.54	4.85	4.96	4.45	2332	0.01
21	KCNH7	Q9NS40	Potassium voltage-gated channel subfamily H member 7	4.85	5.05	5.33	5.31	2313	0.01
22	PDIA4	P13667	Protein disulfide-isomerase A4	4.72	5.23	4.86	4.72	1936	0.01
23	VAT1	Q99536	Synaptic vesicle membrane protein VAT-1 homolog	4.48	4.25	4.79	4.26	1674	0.01
24	FLNC	Q14315	Filamin-C	5.22	4.65	5.22	4.76	688	0.00
25	MRC2	Q9UBG0	C-type mannose receptor 2	4.42	4.62	4.61	4.64	636	0.00
26	ICAM1	P05362	Intercellular adhesion molecule 1	4.18	4.14	4.11	4.04	507	0.00
27	MTMR4	Q9NYA4	Myotubularin-related protein 4	4.31	3.87	4.31	3.99	238	0.00
								5380003	23.88
**intracellular or not annotated**									
1	PROF1	P07737	Profilin-1	5.94	6.35	6.41	6.03	186949	0.83
2	ALDOA	P04075	Fructose-bisphosphate aldolase A	6.13	6.54	6.48	5.85	97474	0.43
3	S10AB	P31949	Protein S100-A11	5.53	5.84	5.84	5.47	84309	0.37
4	HSPB1	P04792	Heat shock protein beta-1	6.03	5.62	6.25	5.55	75441	0.33
5	LDHA	P00338	L-lactate dehydrogenase A chain	5.72	6.37	6.58	5.67	74208	0.33
6	C1S	P09871	Complement C1s subcomponent	5.96	5.74	5.67	6.65	57474	0.26
7	KPYM	P14618	Pyruvate kinase PKM	5.92	6.22	6.52	5.69	43904	0.19
8	TSP2	P35442	Thrombospondin-2	6.53	5.01	5.67	6.39	30600	0.14
9	ANXA5	P08758	Annexin A5	5.67	6.08	6.06	5.37	29399	0.13
10	GSTP1	P09211	Glutathione S-transferase P	5.40	5.45	5.68	5.18	26571	0.12
11	TPM4	P67936	Tropomyosin alpha-4 chain	5.53	5.67	5.76	5.57	20908	0.09
12	SODM	P04179	Superoxide dismutase [Mn], mitochondrial	4.52	5.67	5.48	5.15	19666	0.09
13	TPIS	P60174	Triosephosphate isomerase	5.23	5.73	5.67	5.12	17256	0.08
14	TAGL2	P37802	Transgelin-2	5.08	5.61	5.41	5.19	16718	0.07
15	FLNA	P21333	Filamin-A	6.19	6.44	6.49	6.16	15796	0.07

16	TAGL	Q01995	Transgelin	5.22	5.64	5.39	5.02	13978	0.06
17	GDIR1	P52565	Rho GDP-dissociation inhibitor 1	5.17	5.31	5.31	5.04	13900	0.06
18	PEBP1	P30086	Phosphatidylethanolamine-binding protein 1	4.65	4.80	5.10	4.61	6270	0.03
19	CYC	P99999	Cytochrome c	4.78	4.80	4.51	4.40	5660	0.03
20	TSG6	P98066	Tumor necrosis factor-inducible gene 6 protein	4.73	4.61	4.70	5.25	4747	0.02
21	MYH9	P35579	Myosin-9	5.63	5.67	6.04	5.59	4673	0.02
22	DYH8	Q96JB1	Dynein heavy chain 8, axonemal	5.98	5.82	6.22	6.18	4324	0.02
23	PGK1	P00558	Phosphoglycerate kinase 1	4.70	4.99	5.38	4.39	3576	0.02
24	SH3L1	O75368	SH3 domain-binding glutamic acid-rich-like protein	4.17	4.48	4.48	3.95	2620	0.01
25	TKT	P29401	Transketolase	4.48	5.03	5.04	4.82	2373	0.01
26	LMNA	P02545	Prelamin-A/C [Cleaved into: Lamin-A/C	4.76	3.91	4.36	4.45	663	0.00
27	FLNB	O75369	Filamin-B	4.04	4.32	4.64	4.29	165	0.00
								859620	3.82

Abbreviations: ECM, extracellular matrix; iBAQ value, intensity based absolute quantification; TCA, trichloroacetic acid

Supplemental Table S2: Affymetrix data from human cardiac fibroblasts after stimulation for all expressed cytokines (mean values ≥ 5). Human cardiac fibroblasts derived from four patients were incubated with or without 10 ng/ml TNF-α or 5 ng/ml TGF-β for 24 hours followed by RNA extraction and subsequent gene expression analysis using Affymetrix arrays. Raw data are listed in alphabetic order. Statistical analysis was performed using paired t test.

Gene Symbol	Probeset ID	untreated control, 24 h				TNF-α stimulation, 24h					TGF-β stimulation, 24h				
		Mean #1	Mean #2	Mean #3	Mean #4	Mean #1	Mean #2	Mean #3	Mean #4	p-value	Mean #1	Mean #2	Mean #3	Mean #4	p-value
BMP1	1569001_at	5.06	5.00	5.14	5.20	5.15	4.94	4.86	5.76	0.69	5.40	5.11	5.36	5.00	0.38
BMP1	1569002_x_at	5.43	5.62	5.64	5.24	5.44	5.52	5.03	5.68	0.79	5.54	5.16	5.42	5.36	0.48
BMP1	202701_at	8.37	8.33	9.16	8.91	8.53	8.16	8.90	9.24	0.92	8.61	8.96	9.33	9.21	**0.05**
BMP1	205574_x_at	7.28	7.91	7.60	8.16	7.37	7.06	7.81	8.16	0.60	7.12	8.68	7.84	8.09	0.42
BMP1	206725_x_at	6.32	6.60	6.67	6.92	6.60	6.56	6.52	7.01	0.65	6.53	7.27	6.84	7.06	0.10
BMP1	207595_s_at	6.77	7.10	6.52	7.01	6.41	6.13	6.79	7.16	0.48	6.41	7.57	6.94	7.22	0.40
BMP2	205289_at	8.04	9.46	9.28	9.20	9.76	11.59	10.81	9.50	**0.04**	8.47	9.54	10.96	10.85	0.10
BMP2	205290_s_at	8.09	9.81	9.49	9.39	9.93	11.56	10.98	9.78	**0.03**	8.59	9.88	11.11	11.19	0.10
BMP3	208244_at	3.28	3.53	3.59	3.56	3.85	3.90	3.84	3.74	**0.03**	3.84	3.81	3.57	3.69	0.15
BMP4	211518_s_at	11.75	10.06	8.13	7.69	9.27	9.33	7.57	7.30	0.12	9.76	8.37	7.44	6.77	**0.02**
BMP5	205430_at	2.56	2.70	2.62	2.72	2.71	2.81	2.44	2.83	0.57	2.70	2.36	2.86	2.83	0.80
BMP5	205431_s_at	4.59	5.02	4.47	4.15	4.36	4.12	4.30	4.46	0.39	4.23	4.36	4.27	4.33	0.24
BMP6	206176_at	10.14	9.56	9.92	9.67	10.27	9.41	9.93	9.30	0.46	10.56	10.72	11.35	10.11	**0.04**
BMP6	215042_at	3.82	4.06	3.35	3.83	4.40	3.52	3.92	4.02	0.51	4.04	3.93	4.65	4.18	0.25
BMP6	241141_at	3.93	4.11	4.30	4.26	4.26	3.98	4.26	4.16	0.90	4.26	4.45	4.88	4.47	**0.02**
BMP7	209590_at	3.28	2.74	2.84	2.95	3.24	3.32	2.98	3.18	0.19	3.14	3.36	3.22	3.23	0.17
BMP7	209591_s_at	3.03	2.90	3.22	3.00	3.27	3.04	3.07	3.10	0.41	3.19	3.26	3.43	3.11	**0.03**
BMP7	211259_s_at	5.08	5.47	5.08	5.11	5.17	5.26	5.28	5.48	0.42	5.29	5.25	5.19	5.45	0.42
BMP7	211260_at	5.90	5.76	6.05	5.78	6.44	5.75	5.64	6.11	0.63	6.43	5.95	5.99	6.01	0.16
BMP8A	207866_at	4.56	4.41	4.67	4.52	4.69	4.50	4.51	4.61	0.57	4.61	4.43	4.33	4.47	0.46
BMP8A	220203_at	6.14	5.77	5.90	6.19	5.91	5.88	6.16	6.12	0.87	6.03	6.15	6.12	6.03	0.57
BMP8A	220204_s_at	3.17	3.36	3.35	3.34	3.48	3.39	3.30	3.42	0.31	3.27	3.23	3.32	3.37	0.97
BMP8A /// BMP8B	221615_at	3.68	3.84	3.53	3.94	3.95	3.75	3.70	3.95	0.36	4.28	3.95	3.78	4.17	0.07

BMP8B	207865_s_at	3.32	3.10	3.30	3.40	3.66	3.57	3.19	3.21	0.50	3.45	3.51	3.48	3.53	**0.05**
BMP8B	235275_at	4.67	4.92	4.81	4.67	4.98	4.70	4.70	4.58	0.82	4.52	4.82	5.04	4.73	0.92
BMP10	208292_at	4.85	4.74	4.42	4.61	5.11	4.27	4.65	4.78	0.80	5.09	5.28	4.91	4.92	**0.01**
BMP15	221332_at	5.55	5.39	5.42	5.46	5.76	5.31	5.25	5.27	0.57	5.49	5.15	5.12	5.29	**0.03**
C1QTNF4	223708_at	4.02	3.80	3.98	3.75	3.76	4.16	3.75	3.51	0.58	3.76	3.53	3.71	3.51	**0.00**
CCL1	207533_at	3.84	3.72	3.42	3.67	4.17	3.90	3.45	4.01	0.06	4.04	3.65	3.93	4.24	0.13
CCL2	216598_s_at	12.76	13.35	13.74	13.75	14.03	14.04	14.18	14.12	**0.04**	13.32	13.03	13.71	13.29	0.80
CCL3	205114_s_at	4.75	4.07	4.28	4.57	10.62	6.16	7.80	6.79	**0.03**	5.38	4.08	4.31	6.86	0.26
CCL4	204103_at	4.50	4.29	4.03	4.22	5.55	4.27	4.35	4.82	0.12	4.39	4.60	4.10	4.52	0.26
CCL5	1405_i_at	6.69	4.49	10.21	6.39	11.36	10.46	11.88	10.40	**0.02**	6.20	5.17	9.33	8.01	0.71
CCL5	1555759_a_at	6.85	4.77	10.06	6.59	11.27	9.44	12.01	10.22	**0.01**	6.82	5.28	9.25	8.32	0.56
CCL5	204655_at	6.60	4.17	9.56	6.62	10.76	9.68	11.30	9.70	**0.02**	6.30	5.30	8.76	7.70	0.61
CCL7	208075_s_at	5.00	8.02	8.66	10.79	8.25	9.28	10.10	12.24	**0.03**	5.99	6.35	7.43	8.86	0.24
CCL8	214038_at	5.90	6.67	7.65	9.32	13.33	10.83	11.97	12.81	**0.01**	9.67	5.39	4.60	9.35	0.93
CCL11	210133_at	5.25	9.89	6.52	13.96	9.89	9.64	7.08	14.25	0.33	9.47	10.02	8.14	12.09	0.48
CCL13	206407_s_at	5.45	5.57	5.63	5.68	5.67	6.02	6.23	5.62	0.13	5.46	5.41	5.91	5.35	0.71
CCL13	216714_at	4.02	3.82	3.65	3.75	3.98	3.84	3.96	4.16	0.22	4.11	4.06	3.87	3.98	**0.01**
CCL14 /// CCL15	205392_s_at	4.71	5.00	4.70	4.48	5.15	4.76	5.00	4.95	0.24	5.00	4.82	4.89	4.85	0.27
CCL14 /// CCL15	210390_s_at	2.74	2.94	2.75	2.62	2.88	2.74	2.79	2.59	0.85	2.74	2.73	2.80	2.86	0.87
CCL16	207354_at	3.71	3.57	3.42	3.32	3.59	3.54	3.74	3.43	0.51	3.52	3.46	3.58	3.58	0.80
CCL17	207900_at	5.26	5.44	5.14	5.29	5.51	5.61	5.38	5.39	**0.01**	5.45	5.34	5.47	5.12	0.66
CCL18	209924_at	5.20	4.52	4.61	4.75	5.17	4.62	5.11	4.74	0.35	5.02	4.59	4.59	4.75	0.57
CCL18	32128_at	3.39	3.63	3.67	3.28	3.73	3.65	3.52	3.36	0.52	3.50	3.61	3.58	3.43	0.53
CCL19	210072_at	4.10	4.52	3.97	4.59	4.36	4.51	4.06	6.86	0.32	4.63	4.48	4.38	5.04	0.08
CCL20	205476_at	12.59	9.68	10.62	10.01	13.59	12.98	12.80	12.53	**0.02**	11.56	8.34	9.30	9.81	**0.04**
CCL21	204606_at	5.43	5.62	5.37	5.01	5.38	5.34	5.55	4.83	0.48	5.47	5.72	5.27	5.31	0.39
CCL22	207861_at	5.09	4.80	4.82	4.77	5.10	5.05	5.23	4.82	0.15	5.16	4.98	5.08	4.98	**0.02**
CCL23	210548_at	2.72	2.59	3.23	2.95	3.08	2.67	3.07	3.02	0.49	2.99	2.93	2.54	3.03	1.00

CCL23	210549_s_at	3.32	3.28	3.13	3.42	3.58	3.39	3.45	3.49	**0.05**	3.50	3.25	3.37	3.50	0.15
CCL24	221463_at	5.13	5.28	5.21	4.74	4.85	4.84	4.73	4.71	0.06	4.75	4.99	5.26	4.79	0.29
CCL25	206988_at	3.42	3.51	3.21	3.58	3.82	3.25	3.42	3.28	0.95	3.54	3.45	3.41	3.61	0.29
CCL26	223710_at	7.39	8.22	5.96	7.82	8.45	9.35	7.29	7.98	**0.04**	8.44	8.47	7.29	7.62	0.18
CCL27	207955_at	3.81	4.14	3.73	3.42	3.84	3.74	4.35	3.83	0.52	3.82	3.83	3.97	3.64	0.78
CCL28	224027_at	3.19	2.80	3.90	3.34	4.24	3.28	3.63	3.07	0.50	4.13	2.95	4.05	2.94	0.50
CCL28	224240_s_at	5.36	5.10	5.97	5.38	6.17	5.26	5.56	5.19	0.75	5.80	5.02	5.76	5.09	0.85
CD40LG	207892_at	5.75	5.68	5.71	5.63	5.77	5.85	5.55	5.77	0.61	5.76	5.78	5.61	5.81	0.46
CER1	221378_at	4.09	4.66	4.99	4.50	4.71	5.36	4.75	4.48	0.34	4.65	4.64	4.28	4.54	0.92
CKLF	219161_s_at	9.39	8.37	9.21	8.83	8.98	8.99	9.30	8.29	0.84	9.90	9.11	9.39	9.77	**0.04**
CKLF	221058_s_at	9.11	8.49	9.26	8.72	9.04	9.17	9.25	8.42	0.74	9.70	9.01	9.26	9.55	0.07
CKLF	223451_s_at	9.79	9.30	9.70	9.46	9.61	9.83	9.89	8.95	0.99	10.10	9.84	9.74	10.02	0.06
CLCF1	219500_at	7.83	7.52	8.09	7.23	8.52	7.73	8.08	7.86	0.11	7.81	7.58	8.04	8.19	0.40
CSF1	207082_at	4.99	4.94	5.33	5.23	5.45	5.66	5.82	5.23	0.07	5.23	5.59	4.90	5.23	0.64
CSF1	209716_at	9.33	8.88	9.28	8.60	10.66	10.06	10.38	9.02	**0.02**	9.72	8.25	7.57	8.16	0.26
CSF1	210557_x_at	4.95	5.10	5.17	5.30	5.36	5.48	5.46	5.18	0.15	5.38	5.17	5.12	4.95	0.88
CSF1	211839_s_at	5.78	5.62	5.68	5.71	5.66	5.09	6.17	5.55	0.72	5.68	5.65	5.50	5.71	0.29
CSF2	210228_at	5.56	5.45	5.41	5.25	5.83	5.88	5.50	5.45	**0.04**	5.75	4.79	5.29	5.21	0.44
CSF2	210229_s_at	9.05	7.38	8.48	8.09	12.38	10.21	10.40	9.37	**0.01**	10.30	6.67	7.31	7.83	0.70
CSF3	207442_at	6.83	7.24	9.91	11.18	11.77	9.82	11.70	12.16	0.06	9.45	7.14	8.78	11.16	0.70
CTF1	206813_at	6.03	5.41	5.80	5.95	6.12	5.93	6.39	5.98	0.12	6.03	5.98	6.06	6.08	0.15
CX3CL1	203687_at	5.43	5.12	5.80	4.42	5.34	6.17	5.80	5.34	0.22	4.99	4.16	4.94	5.23	0.44
CX3CL1	823_at	5.03	4.55	6.06	4.87	5.50	6.10	7.00	4.83	0.12	4.69	4.50	5.15	5.14	0.39
CXCL1	204470_at	13.70	13.76	14.08	14.22	14.37	14.08	14.38	14.34	0.05	14.03	13.26	13.68	13.89	0.32
CXCL2	1569203_at	3.52	3.58	3.62	3.98	3.45	3.52	3.48	4.08	0.46	3.28	3.71	3.36	3.59	0.19
CXCL2	209774_x_at	8.41	10.71	11.82	12.03	12.25	12.23	12.77	13.38	0.06	9.46	8.79	9.37	11.90	0.37
CXCL2	230101_at	3.00	3.18	3.35	3.21	3.42	3.22	3.21	3.50	0.30	3.32	3.31	3.35	3.20	0.24
CXCL3	207850_at	8.75	9.25	11.95	11.15	11.98	11.31	12.86	12.66	**0.03**	9.35	7.75	10.08	10.64	0.24

CXCL5	207852_at	4.24	2.71	3.35	6.19	6.79	4.41	4.80	6.68	**0.04**	6.37	4.05	4.20	5.32	0.27
CXCL5	214974_x_at	12.07	10.30	11.68	13.25	13.47	12.04	12.84	13.45	**0.04**	13.05	9.97	11.35	12.38	0.75
CXCL5	215101_s_at	7.92	7.27	5.95	11.81	12.40	6.02	9.11	12.21	0.28	11.92	6.82	6.99	10.57	0.52
CXCL6	206336_at	13.53	13.48	13.48	13.84	13.75	13.48	13.51	13.77	0.51	13.66	13.34	13.40	13.34	0.35
CXCL9	203915_at	4.28	4.64	5.53	4.89	5.23	4.85	5.11	5.14	0.44	5.34	4.72	4.59	5.37	0.71
CXCL10	204533_at	5.17	4.83	4.56	4.76	6.66	9.18	7.63	8.06	**0.01**	5.18	4.84	5.25	5.53	0.17
CXCL11	210163_at	3.01	2.63	3.29	2.52	3.22	4.84	4.46	3.78	0.06	2.34	2.74	2.84	2.74	0.43
CXCL11	211122_s_at	2.74	2.71	3.04	2.78	3.33	5.90	4.26	4.10	0.07	2.69	2.77	2.80	2.98	0.96
CXCL12	203666_at	6.14	8.34	7.05	10.98	8.66	6.56	7.14	9.34	0.85	10.66	9.80	7.67	10.70	0.23
CXCL12	209687_at	9.98	11.34	11.77	13.03	12.15	10.02	12.01	11.92	1.00	13.04	12.10	11.39	12.70	0.40
CXCL13	205242_at	2.81	2.86	2.94	2.72	2.80	2.86	2.80	2.78	0.62	2.76	2.95	3.42	2.77	0.32
CXCL14	218002_s_at	4.61	4.70	4.42	4.25	6.37	4.39	3.94	3.95	0.78	6.07	3.95	4.30	3.93	0.90
CXCL14	222484_s_at	5.14	4.95	4.83	5.00	7.45	5.48	4.95	5.00	0.26	6.48	5.33	5.21	4.87	0.21
CXCL14	237038_at	4.92	4.45	4.63	4.39	4.63	4.73	4.36	4.62	0.94	4.72	4.93	4.32	4.74	0.71
CXCL16	223454_at	5.86	5.72	6.44	5.28	5.66	5.83	6.68	5.62	0.39	5.42	5.19	6.21	5.23	0.06
EBI3	219424_at	5.20	4.69	4.94	5.41	6.89	5.77	5.09	5.96	0.08	5.71	5.44	4.00	5.57	0.76
FAM19A5	229459_at	3.31	2.70	3.35	2.70	2.75	2.88	2.94	2.70	0.34	2.83	2.86	2.92	3.06	0.67
FAM19A5	229655_at	3.54	3.79	3.58	3.25	3.43	3.59	3.84	3.44	0.78	3.55	3.52	3.51	3.34	0.49
FAM19A5	237094_at	2.80	3.47	3.02	2.77	2.97	2.60	2.99	2.61	0.40	3.03	3.50	3.18	2.71	0.26
FAM3B	227194_at	2.98	2.71	3.22	3.08	3.29	2.98	2.90	2.92	0.85	3.23	2.86	3.40	3.47	**0.02**
FASLG	210865_at	4.40	4.88	4.33	4.08	4.58	4.73	4.40	4.44	0.36	4.90	4.81	4.49	4.61	0.14
FASLG	211333_s_at	2.93	3.11	2.88	3.19	3.31	3.55	3.11	3.08	0.15	3.38	3.08	2.87	3.17	0.47
FLT3LG	206980_s_at	4.23	5.69	5.03	5.01	5.23	4.84	5.24	5.01	0.83	5.19	4.93	4.84	5.06	0.97
FLT3LG	210607_at	3.33	4.48	5.63	4.67	4.88	5.31	5.73	5.06	0.11	4.36	4.55	4.12	4.19	0.70
GDF1 /// LASS1	206397_x_at	5.29	5.65	5.46	5.12	4.99	4.86	5.47	5.05	0.21	5.59	5.26	5.28	5.10	0.65
GDF2	221136_at	6.33	6.69	6.19	5.94	6.31	6.39	6.00	5.98	0.23	6.48	6.43	5.97	6.28	0.98
GDF3	220053_at	5.76	5.15	5.60	5.69	5.68	5.60	5.56	5.60	0.68	5.74	5.37	5.71	5.65	0.36
GDF5	206614_at	8.96	8.28	8.11	7.02	6.69	7.78	7.51	6.71	0.14	6.68	6.70	7.19	6.53	**0.04**

GDF9	221314_at	4.59	4.41	4.38	4.52	5.06	4.24	4.40	4.66	0.46	4.93	4.66	4.60	4.82	**0.00**
GPI /// LOC100133951	208308_s_at	10.72	10.89	10.76	10.97	10.64	9.71	10.49	11.06	0.29	10.79	10.66	10.49	11.14	0.60
GDF10	206159_at	3.56	3.31	3.44	3.83	3.38	3.36	3.54	3.46	0.44	3.48	3.43	4.44	3.80	0.39
GDF11	216854_at	5.14	4.96	5.14	4.94	5.39	4.94	4.74	5.13	0.98	5.45	5.33	5.00	5.17	0.19
GDF11	216860_s_at	6.92	7.00	6.84	6.63	6.47	6.63	6.76	6.73	0.22	6.96	6.81	6.63	6.83	0.70
GDF15	221576_at	4.28	3.79	4.02	4.09	4.14	3.96	3.71	4.00	0.41	4.27	3.58	4.09	3.85	0.28
GDF15	221577_x_at	11.87	10.31	8.84	11.23	12.02	10.28	9.53	10.99	0.54	12.09	10.37	8.27	11.58	0.96
GDF15	229868_s_at	4.53	4.59	3.35	4.31	4.12	3.78	3.72	4.19	0.40	4.61	4.44	3.59	4.29	0.72
GREM1	218468_s_at	13.62	12.66	13.43	13.36	13.78	12.75	13.36	13.50	0.23	13.43	11.17	13.10	12.70	0.11
GREM1	218469_at	13.44	12.55	13.05	13.21	13.52	12.57	12.97	13.35	0.46	13.28	10.94	12.73	12.56	0.12
GREM2	220794_at	3.44	3.18	4.12	3.26	3.35	2.91	4.49	3.13	0.83	3.51	2.88	3.93	3.15	0.18
GREM2	235504_at	4.32	4.65	3.98	4.13	4.61	4.26	4.26	4.28	0.64	4.72	4.39	4.28	4.08	0.56
GREM2	240509_s_at	2.87	2.52	2.97	2.34	2.35	2.48	3.11	2.38	0.56	2.31	2.35	3.48	2.30	0.79
GRN	200678_x_at	11.54	11.06	11.72	11.10	11.42	11.00	11.73	11.14	0.40	11.36	11.02	11.30	11.07	0.16
GRN	211284_s_at	10.68	10.26	10.93	10.33	10.72	10.31	10.98	10.49	0.08	10.81	10.36	10.56	10.32	0.75
GRN	216041_x_at	11.05	10.63	11.21	10.67	11.02	10.55	11.20	10.81	0.87	10.99	10.63	10.84	10.62	0.25
IFNA1 /// IFNA13	208344_x_at	4.57	4.79	4.70	4.71	4.89	4.18	4.76	4.84	0.91	4.67	4.69	4.52	4.93	0.92
IFNA1 /// IFNA13	208375_at	3.02	2.20	2.81	2.69	2.76	2.87	2.42	2.68	0.99	2.50	2.75	2.87	2.43	0.87
IFNA2	211338_at	3.51	3.75	3.00	3.16	3.57	3.52	3.27	3.06	1.00	3.52	3.45	3.22	3.35	0.80
IFNA4	207964_x_at	3.88	4.00	3.92	3.77	4.00	3.51	3.98	3.83	0.69	4.28	4.45	3.90	3.92	0.11
IFNA5	214569_at	3.10	2.74	2.74	2.66	2.78	3.35	2.83	2.74	0.58	2.44	3.20	2.67	2.74	0.86
IFNA6	208548_at	3.56	3.48	3.35	3.55	3.70	3.51	3.31	3.34	0.81	3.60	3.53	3.48	3.54	0.18
IFNA7	208259_x_at	4.44	4.37	4.34	3.64	4.21	4.28	3.99	4.17	0.88	4.47	3.92	4.00	3.96	0.59
IFNA8	207932_at	3.20	2.89	2.71	2.81	2.91	3.16	3.18	2.92	0.44	3.10	3.10	3.26	2.83	0.32
IFNA10	208261_x_at	4.02	4.10	3.85	4.12	4.02	3.75	4.12	4.07	0.82	4.21	4.05	4.02	3.83	0.97
IFNA14	208182_x_at	3.54	3.55	3.63	3.67	3.70	3.36	3.85	3.75	0.51	4.15	4.36	3.56	3.71	0.20
IFNA16	208448_x_at	2.89	3.27	3.57	3.22	3.36	3.33	2.83	3.09	0.76	3.28	3.53	2.97	3.37	0.83

IFNA17	211405_x_at	4.32	4.96	4.23	4.86	5.55	4.66	5.11	5.08	0.23	5.72	5.04	4.81	5.16	0.13
IFNA21	211145_x_at	3.62	4.10	3.78	4.18	4.18	3.91	3.64	3.93	0.99	4.29	4.18	3.95	4.02	0.35
IFNB1	208173_at	4.01	4.13	4.09	4.13	4.13	4.03	4.02	4.03	0.53	3.93	4.07	4.16	4.29	0.76
IFNE1	1553574_at	3.49	3.83	3.35	4.62	3.79	4.38	4.14	4.82	**0.04**	3.77	3.97	4.84	4.39	0.34
IFNG	210354_at	4.40	4.11	4.50	4.20	4.69	4.02	4.57	4.36	0.28	4.87	4.58	4.33	4.44	0.19
IFNK	224093_at	3.06	3.46	3.27	2.64	2.96	3.03	3.09	2.74	0.26	2.74	2.81	2.91	2.76	0.15
IFNW1	207817_at	4.24	4.88	4.42	4.44	4.71	4.58	4.47	4.55	0.65	4.67	4.62	4.83	4.56	0.36
IL1A	208200_at	3.88	3.88	4.14	4.34	4.09	3.96	4.27	4.27	0.22	4.05	3.88	4.11	4.18	0.95
IL1A	210118_s_at	8.83	7.96	6.91	10.49	12.15	10.31	10.45	11.61	**0.02**	10.80	8.68	8.75	10.89	0.05
IL1B	205067_at	11.65	11.44	11.98	13.06	14.24	12.97	13.63	13.81	**0.02**	12.88	10.88	11.72	12.97	0.86
IL1B	39402_at	11.37	11.33	11.65	12.74	14.23	12.71	13.45	13.63	**0.03**	12.54	10.87	11.40	12.65	0.82
IL1F10	224262_at	5.32	4.81	5.27	5.03	5.60	5.02	5.22	5.18	0.13	5.53	5.22	5.15	5.29	0.19
IL1F5	222223_s_at	4.11	3.99	3.77	3.92	3.72	4.48	4.43	3.63	0.70	3.69	3.79	4.29	3.63	0.65
IL1F6	221404_at	4.20	4.35	4.05	4.06	4.39	4.01	4.26	4.08	0.88	4.56	5.02	3.97	4.19	0.19
IL1F7	221470_s_at	3.01	3.48	3.60	3.08	2.98	4.18	3.14	3.27	0.72	3.05	2.98	3.15	3.29	0.39
IL1F7	224555_x_at	6.14	6.09	6.10	6.31	6.66	6.52	6.09	6.41	0.13	6.42	6.00	6.34	6.24	0.43
IL1F8	224230_at	3.90	3.75	3.68	4.06	3.69	3.59	3.48	3.66	**0.02**	3.72	4.49	3.62	3.66	0.92
IL1F8	231755_at	3.18	3.54	3.37	3.30	3.85	3.48	3.04	3.41	0.67	3.52	4.05	3.33	3.40	0.16
IL1F9	220322_at	4.35	4.20	4.70	4.55	4.55	4.32	5.15	4.60	0.10	4.54	4.54	4.22	4.18	0.72
IL2	207849_at	2.94	2.95	2.89	2.96	2.75	2.91	2.93	2.96	0.39	2.91	2.59	2.76	2.91	0.15
IL3	207906_at	3.35	3.51	3.45	3.55	3.72	3.77	3.45	3.99	0.07	3.91	3.84	3.48	3.55	0.17
IL4	207538_at	3.05	2.95	2.52	3.04	3.13	3.62	3.05	2.94	0.20	2.95	2.54	2.74	2.81	0.42
IL4	207539_s_at	2.88	2.33	2.89	2.75	2.74	2.74	2.75	2.81	0.75	2.82	2.90	2.49	2.83	0.84
IL5	207952_at	3.32	2.80	2.89	2.75	2.62	2.61	2.86	2.57	0.16	2.77	2.97	3.01	2.37	0.43
IL6	205207_at	12.43	13.07	13.25	13.61	13.79	13.65	13.84	14.19	**0.03**	13.25	13.12	13.49	13.84	0.14
IL7	206693_at	4.84	5.42	5.62	5.28	5.63	6.23	6.59	7.06	**0.02**	4.36	3.60	4.01	4.79	0.05
IL8	202859_x_at	14.08	13.89	14.26	14.25	14.53	14.30	14.40	14.47	**0.02**	14.21	13.75	14.22	14.18	0.63
IL8	211506_s_at	11.93	11.75	11.64	13.38	14.05	11.52	13.42	13.79	0.16	13.53	11.48	12.82	13.32	0.27

IL9	208193_at	2.97	2.90	2.96	2.80	3.09	3.18	3.13	2.75	0.15	3.03	3.23	2.84	3.11	0.27
IL10	207433_at	3.19	3.07	3.23	3.17	3.31	2.99	3.22	3.30	0.51	3.05	2.78	3.29	3.19	0.36
IL11	206924_at	4.50	5.25	8.71	10.96	11.11	6.83	9.98	12.96	0.11	10.42	11.36	12.56	13.22	**0.02**
IL11	206926_s_at	5.34	5.68	6.31	7.79	8.03	5.68	7.37	9.90	0.09	7.57	8.71	10.73	10.34	**0.01**
IL12A	207160_at	5.43	4.03	5.78	3.85	4.62	4.88	4.92	4.04	0.73	4.02	4.02	5.72	3.80	0.34
IL12B	207901_at	2.61	2.61	2.45	2.68	2.64	2.84	3.01	2.64	0.23	2.53	2.71	2.44	2.52	0.56
IL13	207844_at	5.67	5.94	5.89	5.72	5.54	5.51	6.05	5.45	0.26	5.70	5.71	5.85	5.53	0.17
IL15	205992_s_at	7.42	7.33	9.28	8.27	8.70	9.48	9.63	9.75	**0.04**	7.29	7.41	9.03	8.50	0.89
IL15	217371_s_at	5.43	4.54	6.61	6.50	6.35	7.01	7.30	7.50	0.05	4.98	5.24	7.06	6.48	0.56
IL16	1555016_at	4.67	4.81	4.88	4.77	5.41	4.90	4.67	4.98	0.37	4.93	5.15	4.89	5.18	0.06
IL16	209827_s_at	4.59	4.58	4.92	4.65	4.61	4.76	4.97	5.01	0.14	4.91	5.10	5.15	5.01	**0.01**
IL16	209828_s_at	4.19	4.28	4.30	4.38	4.40	4.90	4.23	4.03	0.65	4.28	4.23	4.07	4.01	0.26
IL17A	208402_at	3.22	2.62	3.00	3.00	2.84	2.74	3.23	2.71	0.63	3.11	2.94	2.96	3.07	0.55
IL17A	216876_s_at	3.06	2.71	2.65	2.68	2.73	2.94	2.50	2.61	0.54	2.79	2.62	2.78	2.53	0.34
IL17B	220273_at	4.42	5.47	5.28	4.93	5.46	5.11	5.68	5.10	0.36	5.68	5.57	5.62	5.36	0.13
IL17C	224079_at	6.17	5.97	6.11	5.88	6.06	6.11	5.97	6.18	0.69	6.09	6.02	5.98	6.14	0.82
IL17D	227401_at	6.66	6.35	8.27	4.79	5.14	6.71	7.43	4.87	0.35	5.13	5.73	7.34	4.45	**0.04**
IL17F	234408_at	4.23	4.26	3.91	3.80	3.88	4.11	3.67	4.10	0.49	4.19	3.91	3.55	3.86	0.20
IL18	206295_at	7.42	4.64	3.63	2.75	3.68	5.24	2.90	2.91	0.41	3.11	3.38	2.60	2.98	0.20
IL19	220745_at	4.92	5.13	4.83	6.17	5.42	5.85	6.16	5.87	0.19	5.36	4.39	4.76	5.36	0.39
IL20	224071_at	4.30	4.69	4.29	4.08	4.51	3.84	4.18	4.49	0.78	4.79	4.43	4.61	4.64	0.23
IL21	221271_at	4.30	4.15	3.84	4.49	4.91	4.26	4.14	4.27	0.34	4.09	4.28	4.36	4.46	0.56
IL22	221165_s_at	4.61	4.31	4.50	4.55	4.60	4.48	4.27	4.46	0.67	4.51	4.64	4.42	4.53	0.76
IL22	222974_at	2.83	2.51	2.97	2.68	2.49	3.05	2.46	2.66	0.73	2.55	2.98	2.42	2.82	0.81
IL23A	220054_at	5.08	5.31	4.97	5.08	5.35	5.52	5.34	6.45	0.13	5.43	5.34	5.21	5.44	**0.05**
IL24	206569_at	5.01	4.49	7.16	9.65	7.41	7.58	9.00	10.53	**0.02**	5.07	4.57	4.49	7.13	0.20
IL25	220971_at	5.65	5.80	5.76	5.31	5.36	5.44	5.31	5.48	0.20	5.45	5.42	5.54	5.27	0.06
IL26	221111_at	3.60	4.88	7.07	2.99	2.75	5.32	5.71	2.72	0.28	2.71	2.74	4.67	2.83	0.08

IL27	1552995_at	5.61	5.37	5.49	5.48	5.92	5.55	5.74	5.50	0.06	5.62	5.46	5.18	5.55	0.73
IL28A	1552915_at	4.20	4.02	4.26	3.96	4.25	3.83	3.98	4.14	0.63	4.34	4.02	3.75	4.16	0.81
IL28A /// IL28B	1552609_s_at	3.69	3.43	3.40	3.71	3.69	3.57	3.64	3.62	0.39	4.00	3.54	3.66	3.65	0.16
IL29	1552917_at	4.29	4.52	4.75	4.33	4.50	4.45	4.51	4.50	0.90	4.66	4.38	4.50	4.50	0.81
IL32	203828_s_at	7.33	8.16	8.15	8.51	8.75	9.56	9.19	8.81	**0.03**	8.02	9.02	8.01	8.54	0.24
IL33	209821_at	9.57	10.71	12.19	11.25	11.92	11.86	12.12	11.30	0.22	11.27	10.64	12.68	11.35	0.26
LASS1	229448_at	5.20	5.32	4.78	4.94	5.38	5.32	5.24	5.07	0.14	5.53	5.54	5.00	5.26	**0.00**
LEFTY1	206268_at	4.07	4.27	3.71	4.53	4.48	4.26	4.05	4.24	0.55	5.02	6.21	3.85	5.58	0.07
LEFTY2	206012_at	4.87	4.52	4.65	5.07	4.93	4.77	4.92	5.14	0.07	4.83	5.03	5.06	5.08	0.22
LIF	205266_at	9.28	8.88	10.51	10.70	11.46	10.05	11.19	12.01	**0.02**	10.33	9.85	11.15	11.92	**0.00**
LTA	206975_at	4.36	4.54	4.66	4.25	4.84	4.42	4.79	4.39	0.29	5.04	4.64	4.51	4.52	0.28
MIF	217871_s_at	13.67	13.16	13.65	13.36	13.50	13.55	13.54	13.39	0.80	13.61	13.10	13.51	13.56	0.81
MSTN	207145_at	2.99	2.32	2.95	2.40	2.27	2.66	2.42	2.25	0.34	2.22	2.29	2.54	2.69	0.39
NAMPT	1555167_s_at	5.91	7.50	6.46	8.21	8.31	6.02	7.54	9.09	0.44	7.77	7.33	5.70	8.81	0.54
NAMPT	217738_at	8.40	9.83	9.70	10.60	10.57	9.97	10.34	11.46	0.12	9.71	9.64	8.30	11.07	0.94
NAMPT	217739_s_at	8.96	10.67	10.43	11.05	11.14	11.15	11.24	12.12	0.06	10.24	10.42	8.55	11.33	0.84
NAMPT	243296_at	3.71	5.61	6.20	7.05	6.36	6.64	7.04	7.99	0.05	5.28	5.64	4.22	7.00	0.89
NODAL	230916_at	6.15	5.63	5.77	5.64	6.10	5.99	5.85	5.95	0.16	5.99	5.71	5.85	5.98	0.47
NODAL	237896_at	4.01	4.05	3.87	4.12	4.46	3.74	3.95	4.30	0.57	4.33	4.08	4.12	4.28	0.06
OSM	214637_at	5.56	5.38	5.97	5.42	5.91	5.48	5.76	5.47	0.58	5.73	5.60	5.66	5.43	0.88
OSM	230170_at	3.29	3.48	2.79	3.00	3.38	3.27	3.57	3.45	0.29	3.41	3.50	3.49	3.50	0.13
PF4	206390_x_at	7.20	6.93	8.20	7.59	6.98	7.30	8.91	7.56	0.39	7.01	6.39	7.60	7.27	**0.02**
PF4V1	207815_at	8.26	7.71	6.98	9.46	8.22	8.19	7.29	8.69	0.99	8.19	6.31	4.80	7.84	0.06
PPBP	214146_s_at	3.40	2.99	3.34	3.82	4.91	3.45	3.61	4.96	0.06	4.84	3.39	3.17	3.92	0.30
SCGB3A1	230378_at	5.28	5.64	5.43	5.71	5.71	5.64	5.38	5.76	0.40	5.93	5.86	5.32	5.51	0.52
SCYE1	202541_at	10.00	8.80	8.47	8.40	8.71	9.26	8.66	8.30	0.66	8.67	8.77	8.66	8.48	0.50
SCYE1	202542_s_at	10.55	9.75	9.61	9.94	9.71	10.08	9.74	9.88	0.69	9.79	9.58	9.74	10.12	0.52
SCYE1	227605_at	8.68	7.49	7.40	7.33	7.64	7.64	7.34	7.25	0.40	7.46	7.64	7.60	7.30	0.55

SLURP1	214536_at	5.43	5.86	5.66	5.40	5.74	5.21	5.50	5.34	0.52	5.55	5.43	5.46	5.22	0.22
SPP1	1568574_x_at	3.19	2.85	2.62	2.65	2.89	3.01	2.50	2.72	0.69	2.92	2.79	2.69	2.49	0.24
SPP1	209875_s_at	5.43	3.29	4.28	2.98	5.19	3.24	5.31	2.97	0.57	5.18	3.49	4.57	3.62	0.32
THNSL2	219044_at	7.68	6.56	6.94	5.12	6.92	6.93	7.01	5.28	0.88	6.79	6.62	6.69	5.49	0.56
THNSL2	239949_at	3.97	4.04	4.33	4.35	4.33	4.06	4.27	4.12	0.86	4.53	4.03	4.08	3.99	0.95
THPO	211154_at	3.11	3.25	3.27	3.24	3.20	4.00	3.17	3.13	0.50	3.14	3.41	3.36	2.97	0.99
THPO	211155_s_at	2.82	2.58	3.20	2.82	2.69	3.02	2.69	2.75	0.75	2.76	2.65	3.08	2.82	0.56
THPO	211831_s_at	4.28	3.68	4.00	4.13	4.27	4.08	3.85	4.03	0.81	4.14	4.19	4.18	4.10	0.44
TNF	207113_s_at	4.41	4.45	4.41	4.27	4.44	4.95	4.51	4.49	0.13	4.25	4.47	4.15	4.51	0.76
TNFSF10	202687_s_at	6.86	7.61	6.82	8.25	9.11	8.85	8.50	8.53	**0.05**	8.30	6.33	5.46	7.78	0.57
TNFSF10	202688_at	7.46	7.74	7.21	7.80	8.64	8.85	8.72	8.23	**0.02**	7.60	6.10	5.59	7.65	0.18
TNFSF10	214329_x_at	7.41	8.00	7.68	8.32	9.22	9.39	9.24	8.66	**0.03**	8.35	6.32	6.24	7.60	0.31
TNFSF11	210643_at	2.43	2.54	3.17	2.54	2.51	2.41	3.00	2.44	0.24	2.57	2.46	3.16	4.41	0.38
TNFSF11	211153_s_at	3.07	3.01	3.03	3.07	3.09	2.79	3.31	2.94	0.91	3.16	2.95	3.30	3.35	0.16
TNFSF12	205611_at	5.67	6.44	6.52	5.64	5.73	6.61	6.34	5.59	0.98	5.73	6.02	5.95	5.22	0.09
TNFSF12 /// TNFSF13	209499_x_at	5.91	5.55	5.60	5.57	5.80	5.14	5.50	5.52	0.14	5.90	5.74	5.25	5.48	0.61
TNFSF12 /// TNFSF13	209500_x_at	7.08	6.74	6.66	6.57	7.42	6.85	6.99	6.57	0.10	7.23	6.73	6.32	6.85	0.90
TNFSF12 /// TNFSF13	211495_x_at	5.55	5.62	5.67	5.51	6.04	5.19	5.53	5.61	0.98	5.94	5.72	5.18	5.74	0.79
TNFSF13	210314_x_at	6.81	6.68	6.08	6.26	7.20	6.35	6.75	6.29	0.45	6.97	6.11	6.24	5.71	0.40
TNFSF13B	223501_at	5.53	5.67	7.07	5.18	5.57	7.70	7.22	6.85	0.15	4.65	4.34	5.67	5.21	0.07
TNFSF13B	223502_s_at	5.35	5.33	7.03	5.09	5.42	7.79	7.27	6.84	0.15	4.96	4.62	5.96	5.21	0.14
TNFSF14	207907_at	4.90	5.73	5.12	5.74	5.63	5.58	4.81	5.64	0.86	5.63	5.31	4.39	4.85	0.43
TNFSF15	221085_at	3.40	3.26	3.84	3.16	3.39	3.49	4.07	3.13	0.24	3.23	3.39	3.21	3.19	0.41
TRBC1 /// TRBV19	217326_x_at	4.84	5.18	4.83	5.15	5.09	4.79	4.86	5.25	1.00	5.18	5.31	5.02	5.29	**0.03**
TSLP	235737_at	7.13	8.21	10.28	8.75	7.30	9.91	10.88	8.72	0.21	6.17	7.02	8.05	8.74	0.10
UNQ464	236981_at	3.02	3.25	2.97	3.37	3.15	3.08	3.15	3.18	0.90	3.09	2.72	2.99	3.25	0.38
VSTM1	235818_at	3.71	4.18	3.89	3.87	4.10	3.99	4.04	4.00	0.39	3.82	3.79	3.89	3.88	0.59
XCL1	206365_at	2.74	2.73	2.64	2.60	2.73	2.92	2.58	2.69	0.43	2.76	2.73	2.80	2.69	0.17

XCL1	206366_x_at	4.70	4.73	4.67	4.88	5.17	5.16	4.90	4.94	0.05	5.04	4.68	4.50	4.71	0.93
XCL1 /// XCL2	214567_s_at	3.66	3.77	3.71	3.78	3.84	3.30	3.61	3.53	0.32	3.77	3.66	3.64	3.35	0.35

Supplemental Table S3: Affymetrix data from human cardiac fibroblasts after stimulation for all expressed collagens. Human cardiac fibroblasts derived from four patients were incubated with or without 10 ng/ml TNF-α or 5 ng/ml TGF-β for 24 hours followed by RNA extraction and subsequent gene expression analysis using Affymetrix arrays. Raw data are listed in alphabetic order. Statistical analysis was performed using paired t test.

Gene Symbol	Probeset ID	untreated control, 24 h				TNF-α stimulation, 24h					TGF-β stimulation, 24h				
		Mean #1	Mean #2	Mean #3	Mean #4	Mean #1	Mean #2	Mean #3	Mean #4	p-value	Mean #1	Mean #2	Mean #3	Mean #4	p-value
COL1A1	1556499_s_at	13.80	14.02	13.94	13.98	13.80	13.93	13.87	13.92	0.07	13.92	14.05	14.02	13.78	0.94
COL1A1	202310_s_at	11.72	13.27	13.00	13.30	12.24	12.02	12.68	13.12	0.46	12.60	13.48	13.25	13.36	0.15
COL1A1	202311_s_at	10.84	11.74	11.64	11.88	10.61	10.99	11.17	11.47	**0.02**	11.44	12.19	12.11	11.94	**0.04**
COL1A1	202312_s_at	5.68	5.42	5.48	5.49	5.59	5.71	5.56	5.27	0.90	5.58	5.72	5.38	5.54	0.71
COL1A1	217430_x_at	7.92	8.95	8.85	9.04	8.44	8.11	8.52	9.10	0.64	8.56	9.68	8.92	8.99	0.17
COL1A2	202403_s_at	14.23	14.36	14.33	14.40	14.36	14.10	14.28	14.43	0.69	14.41	14.45	14.38	14.45	0.06
COL1A2	202404_s_at	14.20	14.15	14.17	14.26	14.15	14.10	14.12	14.20	**0.00**	14.21	14.24	14.26	14.17	0.57
COL1A2	229218_at	9.61	10.20	10.99	11.02	10.47	10.19	10.78	10.87	0.65	10.73	10.64	11.19	10.58	0.38
COL3A1	201852_x_at	12.80	13.55	13.70	13.99	13.51	12.32	13.62	14.02	0.74	13.55	13.76	13.89	14.08	0.13
COL3A1	211161_s_at	12.83	13.49	13.64	13.68	13.13	13.36	13.48	13.69	0.96	13.22	13.58	13.60	13.56	0.52
COL3A1	215076_s_at	13.68	14.17	14.37	14.43	14.11	13.95	14.32	14.48	0.73	14.13	14.33	14.37	14.47	0.22
COL3A1	232458_at	5.93	7.86	9.65	9.13	7.35	8.49	9.28	9.30	0.31	7.33	7.85	9.16	8.69	0.81
COL4A1	211980_at	13.31	13.42	12.27	13.41	13.30	13.17	12.12	13.25	0.07	13.55	13.71	13.25	13.52	0.13
COL4A1	211981_at	10.53	10.85	8.43	11.41	11.10	9.58	8.48	11.22	0.62	11.80	11.37	10.12	11.78	0.05
COL4A2	211964_at	13.47	13.45	12.64	13.48	13.36	13.19	12.42	13.52	0.14	13.51	13.76	13.33	13.58	0.15
COL4A2	211966_at	10.37	10.75	8.39	9.60	10.78	9.40	8.39	9.35	0.49	11.24	11.18	9.56	9.76	0.06
COL4A3	214641_at	6.62	5.32	4.92	4.84	4.93	5.63	4.08	4.63	0.25	5.06	6.22	4.87	5.17	0.87
COL4A3	216367_at	3.63	3.42	3.66	3.43	3.42	3.22	3.31	3.50	0.15	3.42	3.16	3.79	3.53	0.60
COL4A3	216368_s_at	3.75	3.12	3.33	3.50	3.02	3.04	3.59	3.22	0.39	3.18	3.72	2.80	3.65	0.77
COL4A3	216893_s_at	3.45	3.02	2.81	3.13	3.06	2.84	2.93	2.93	0.23	3.00	3.42	2.61	2.95	0.60
COL4A3	216896_at	3.32	3.29	2.70	2.70	2.72	2.89	2.62	2.58	0.09	2.93	2.53	3.09	2.80	0.56
COL4A3	216898_s_at	4.71	4.20	3.68	4.18	4.37	3.52	3.89	4.09	0.32	4.38	4.32	4.20	4.01	0.87
COL4A3	222073_at	9.40	8.50	7.51	6.79	7.17	8.78	7.12	6.90	0.40	7.50	8.86	7.56	7.26	0.68

COL4A4	214602_at	9.86	9.89	8.97	7.81	8.37	9.83	8.92	8.10	0.47	8.95	10.35	8.70	8.58	0.97
COL4A4	229779_at	11.01	10.50	9.79	9.08	9.20	10.69	9.42	9.02	0.34	9.79	11.07	9.81	9.45	0.89
COL4A4	241565_at	4.19	4.19	4.27	3.94	4.15	4.15	3.91	4.21	0.75	4.15	4.29	4.12	4.22	0.65
COL4A5	213110_s_at	11.07	9.08	8.91	9.40	9.69	8.71	9.11	9.09	0.25	9.86	8.88	7.90	8.61	**0.04**
COL4A5	234387_at	3.41	3.78	3.48	3.32	3.08	3.33	3.92	3.62	0.97	3.29	3.63	3.18	3.49	0.39
COL4A6	210945_at	3.74	3.30	2.93	3.10	3.07	2.73	2.98	3.24	0.30	3.31	3.16	3.20	3.11	0.67
COL4A6	211473_s_at	3.76	3.08	3.43	3.16	3.36	3.52	3.10	3.35	0.91	3.59	3.57	3.54	3.35	0.33
COL4A6	213992_at	7.61	3.62	4.44	3.15	4.57	3.45	5.21	3.54	0.59	4.59	3.07	4.18	3.21	0.27
COL5A1	203325_s_at	10.85	11.76	11.92	11.76	11.15	11.19	11.47	11.72	0.41	11.58	12.37	12.52	12.21	**0.00**
COL5A1	212488_at	11.12	11.89	11.68	11.90	11.55	10.95	11.18	11.94	0.48	11.85	12.63	12.59	12.52	**0.00**
COL5A1	212489_at	10.60	10.98	10.83	11.53	10.98	9.69	10.57	11.44	0.44	11.52	11.90	11.77	11.95	**0.01**
COL5A2	221729_at	12.84	12.99	12.60	13.34	12.46	13.01	12.43	13.39	0.31	12.61	13.22	13.12	13.37	0.46
COL5A2	221730_at	12.04	12.29	11.54	12.63	11.84	11.43	11.50	12.81	0.38	11.91	12.58	12.18	12.79	0.23
COL5A3	218975_at	5.76	6.19	7.41	6.02	5.78	6.06	7.17	6.32	0.92	5.80	6.25	7.18	6.28	0.76
COL5A3	52255_s_at	5.97	6.71	8.00	6.24	6.23	6.74	7.60	6.54	0.80	5.72	6.54	7.92	6.94	0.85
COL6A1	212091_s_at	6.84	7.96	7.76	8.53	7.81	7.14	7.83	8.27	0.98	7.77	7.82	7.63	8.04	0.89
COL6A1	212937_s_at	6.25	7.65	7.02	7.41	6.28	6.11	6.70	7.47	0.32	6.56	7.22	6.75	6.86	0.30
COL6A1	212938_at	5.55	6.46	5.95	6.58	5.60	5.50	6.03	6.36	0.36	5.90	6.42	6.07	5.89	0.79
COL6A1	212939_at	4.12	3.83	4.03	4.08	4.07	3.99	4.20	4.10	0.23	3.95	3.88	4.00	4.27	0.88
COL6A1	212940_at	7.08	9.71	8.85	9.87	7.87	7.57	8.56	9.90	0.56	8.42	9.30	8.82	9.11	0.95
COL6A1	213428_s_at	12.77	13.39	13.60	13.67	13.14	13.09	13.46	13.62	0.83	13.07	13.28	13.46	13.39	0.68
COL6A1	214200_s_at	3.62	3.97	3.67	4.10	3.80	3.84	4.43	4.18	0.33	4.10	4.23	3.47	4.18	0.36
COL6A1	216904_at	4.18	4.66	4.21	4.67	4.47	4.51	4.39	4.56	0.64	4.80	4.91	3.88	4.38	0.79
COL6A2	209156_s_at	9.53	10.67	10.71	11.72	10.54	9.21	10.55	11.36	0.66	10.37	10.60	10.24	11.13	0.84
COL6A2	213290_at	8.20	8.59	9.34	9.25	8.36	8.49	9.41	8.98	0.75	8.42	8.53	8.99	9.03	0.46
COL6A3	201438_at	10.97	13.26	13.26	13.53	12.06	13.03	12.74	13.31	0.94	12.86	13.54	13.41	13.50	0.29
COL7A1	204136_at	4.75	5.55	6.88	7.74	6.58	6.25	6.91	8.76	0.10	6.95	7.79	8.59	9.07	**0.00**
COL7A1	217312_s_at	7.34	7.28	7.88	8.57	7.89	7.37	8.16	9.33	0.07	7.93	8.63	9.29	9.50	**0.01**

COL8A1	214587_at	8.91	7.82	5.33	7.92	9.50	5.31	6.49	7.88	0.82	9.43	7.76	6.83	7.76	0.32
COL8A1	221152_at	3.32	3.16	2.76	2.98	3.43	3.24	3.31	3.08	0.16	2.97	2.58	2.97	2.96	0.36
COL8A2	221900_at	4.25	5.10	4.42	4.89	4.43	4.65	3.72	4.44	0.15	5.30	5.53	4.17	5.81	0.16
COL8A2	52651_at	4.90	4.99	4.85	5.03	5.21	5.11	4.76	5.14	0.26	5.53	5.34	4.96	5.55	**0.04**
COL9A2	213622_at	5.46	5.01	5.43	5.26	5.19	5.41	5.24	5.32	0.98	5.12	4.84	5.52	5.08	0.19
COL9A2	232542_at	4.70	4.97	4.81	4.44	4.81	4.39	4.68	4.59	0.55	4.94	4.48	4.68	4.56	0.72
COL10A1	205941_s_at	5.02	5.06	5.34	6.51	8.07	4.92	5.38	6.36	0.44	9.71	8.00	7.70	8.97	**0.01**
COL10A1	217428_s_at	4.21	4.67	4.15	6.38	7.82	4.93	4.09	5.79	0.46	9.78	7.36	7.47	9.06	**0.01**
COL11A1	204320_at	5.79	4.86	4.21	5.66	5.28	4.86	4.60	4.84	0.45	6.38	5.52	3.69	6.09	0.37
COL11A1	229271_x_at	3.48	2.73	2.47	2.78	2.59	2.62	2.75	2.57	0.42	3.13	3.53	2.37	3.12	0.53
COL11A1	37892_at	6.49	5.16	4.03	5.99	5.46	5.26	3.48	5.13	0.10	6.72	6.27	3.63	6.56	0.32
COL11A2	213870_at	5.53	5.15	5.26	5.53	5.64	5.56	5.48	5.69	**0.04**	5.67	5.53	5.42	5.81	**0.02**
COL11A2	216993_s_at	6.20	5.84	6.35	6.03	6.36	6.16	6.16	6.29	0.31	6.33	6.17	6.40	6.45	0.07
COL12A1	225664_at	9.42	10.82	10.27	11.55	10.16	10.73	9.62	11.83	0.82	10.68	11.18	10.62	11.83	0.10
COL12A1	231766_s_at	6.34	7.95	6.65	9.41	7.64	7.61	6.31	9.41	0.72	8.37	8.70	6.94	10.06	0.09
COL12A1	231879_at	4.98	6.44	4.58	8.39	7.04	4.58	4.92	8.20	0.92	8.06	7.29	6.17	8.85	0.08
COL12A1	233109_at	3.07	3.46	3.48	3.92	3.11	2.98	3.36	3.91	0.31	3.34	4.02	3.75	3.71	0.26
COL12A1	234951_s_at	4.67	4.84	4.68	6.09	4.95	4.51	4.86	6.00	0.95	5.30	5.31	4.58	6.01	0.30
COL15A1	203477_at	4.51	4.34	9.44	5.83	3.86	4.38	8.73	5.62	0.12	5.73	6.18	10.49	8.23	**0.01**
COL16A1	204345_at	10.23	10.83	10.18	12.02	10.91	10.71	10.30	12.25	0.27	10.97	11.25	10.41	11.85	0.21
COL18A1	209081_s_at	6.80	7.61	7.17	8.42	6.51	7.77	6.80	8.48	0.48	6.86	7.91	7.40	8.54	**0.05**
COL18A1	209082_s_at	6.98	8.17	7.45	8.78	7.12	8.37	7.17	8.81	0.86	7.30	8.33	7.51	8.79	0.14
COL20A1	232638_at	2.67	2.79	3.08	2.84	2.94	3.08	3.03	2.84	0.24	2.78	2.70	2.94	2.87	0.72
COL20A1	232733_s_at	5.20	4.78	4.68	5.07	5.31	4.88	4.80	5.20	**0.00**	5.54	5.40	4.75	5.08	0.16
COL21A1	208096_s_at	5.88	4.83	5.19	4.80	4.52	4.90	4.74	4.67	0.24	4.81	3.74	4.83	4.79	0.10
COL22A1	228873_at	4.21	4.38	7.07	5.09	5.05	4.54	7.00	4.41	0.86	5.04	5.92	9.63	5.01	0.12
COL22A1	243301_at	3.08	2.82	3.18	2.70	2.84	3.09	3.93	2.75	0.40	2.86	2.91	4.86	3.29	0.29
COL27A1	1564008_at	4.75	4.73	4.87	4.78	5.14	4.58	4.75	5.00	0.57	5.13	5.11	4.75	4.73	0.36

COL27A1	225288_at	4.49	4.45	5.11	6.40	6.14	4.39	4.81	6.38	0.53	6.39	5.89	6.20	7.27	**0.01**
COL27A1	225292_at	5.73	5.86	6.03	6.42	6.34	6.13	5.71	6.64	0.39	6.88	6.78	6.43	6.96	**0.02**
COL27A1	225293_at	6.43	6.59	6.45	7.66	7.43	7.51	6.49	7.91	0.10	7.84	7.69	7.92	8.46	**0.00**
COL27A1	230603_at	4.95	4.74	5.04	4.81	5.13	4.66	4.86	4.54	0.46	4.89	4.86	4.77	4.92	0.84

Supplemental Table S4: Affymetrix data from human cardiac fibroblasts after stimulation for all expressed MMPs and TIMPs. Human cardiac fibroblasts derived from four patients were incubated with or without 10 ng/ml TNF-α or 5 ng/ml TGF-β for 24 hours followed by RNA extraction and subsequent gene expression analysis using Affymetrix arrays. Raw data are listed in alphabetic order. Statistical analysis was performed using paired t test.

Gene Symbol	Probeset ID	untreated control, 24 h				TNF-α stimulation, 24h					TGF-β stimulation, 24h				
		Mean #1	Mean #2	Mean #3	Mean #4	Mean #1	Mean #2	Mean #3	Mean #4	p-value	Mean #1	Mean #2	Mean #3	Mean #4	p-value
MMP1	204475_at	12.84	12.61	13.33	13.58	13.77	12.98	13.84	13.23	0.26	13.45	12.72	12.74	13.08	0.76
MMP2	1566677_at	3.02	3.15	3.22	2.97	2.93	3.06	2.99	3.08	0.35	2.99	3.06	2.95	3.01	0.27
MMP2	1566678_at	4.65	4.67	4.78	4.73	4.80	4.67	4.91	4.97	0.07	4.74	4.89	4.98	5.25	0.07
MMP2	201069_at	12.25	12.82	12.57	13.52	13.10	12.68	12.47	13.53	0.55	13.06	13.19	12.57	13.30	0.36
MMP3	205828_at	8.11	5.29	7.97	9.20	11.46	6.87	9.73	9.58	0.06	10.18	5.09	8.97	9.49	0.21
MMP8	207329_at	3.98	3.76	3.86	5.79	4.54	4.08	3.67	6.40	0.17	4.19	3.97	3.68	5.28	0.72
MMP9	203936_s_at	5.20	4.92	5.14	5.27	5.20	5.20	5.21	5.34	0.19	5.28	4.38	5.37	5.23	0.70
MMP10	205680_at	6.57	5.72	5.92	8.00	8.28	6.75	7.77	7.34	0.19	7.16	6.62	8.70	9.70	0.06
MMP11	203876_s_at	5.06	5.36	4.79	4.88	4.81	4.82	4.93	4.93	0.41	4.95	4.71	4.64	4.84	0.19
MMP11	203877_at	4.16	3.93	4.43	4.26	4.31	4.18	4.36	4.03	0.84	4.54	4.27	4.29	4.44	0.20
MMP11	203878_s_at	6.21	6.59	5.46	6.11	5.96	6.02	5.97	5.60	0.47	6.08	6.54	6.09	6.20	0.49
MMP11	235908_at	5.62	6.08	5.31	5.54	5.73	5.88	5.98	5.37	0.65	5.79	5.49	5.80	5.71	0.81
MMP12	204580_at	9.83	5.56	6.47	3.62	10.20	8.41	8.05	4.82	0.06	7.91	5.62	6.39	4.14	0.56
MMP16	207012_at	3.43	5.65	4.57	5.03	3.67	5.06	4.71	4.82	0.62	4.15	6.24	4.57	5.01	0.20
MMP16	207013_s_at	4.92	5.16	4.76	4.84	4.69	4.82	4.63	4.79	0.05	4.89	5.26	4.55	4.84	0.62
MMP16	208166_at	3.32	3.53	3.35	3.26	3.32	3.09	3.28	3.32	0.39	3.27	3.68	3.16	3.33	0.92
MMP16	208167_s_at	3.34	4.23	3.77	3.80	3.56	3.79	3.93	3.63	0.75	3.84	3.67	3.76	4.13	0.80
MMP17	206234_s_at	5.73	5.12	5.94	5.85	5.55	5.74	5.74	5.87	0.76	5.69	5.58	5.76	5.69	0.89
MMP19	204574_s_at	6.34	6.33	6.10	6.04	6.24	6.27	6.19	5.94	0.41	6.13	6.28	6.21	6.25	0.86
MMP19	204575_s_at	7.24	7.82	7.26	7.90	7.12	7.75	7.39	7.92	0.87	7.01	8.14	7.60	7.62	0.84
MMP24	208387_s_at	3.33	3.60	4.01	3.66	3.95	3.41	3.94	3.75	0.58	3.80	4.23	3.72	3.66	0.41
MMP24	213171_s_at	6.05	5.46	5.74	5.36	5.62	5.72	5.60	5.19	0.45	5.83	5.73	5.29	5.51	0.72
MMP25	207289_at	5.01	5.51	5.05	4.87	4.97	4.88	5.24	4.90	0.58	4.94	5.21	4.58	5.11	0.41

MMP25	207890_s_at	5.43	5.57	5.40	5.43	5.55	5.74	5.36	5.57	0.12	5.36	5.60	5.62	5.55	0.31
MMP28	219909_at	3.09	3.27	3.87	3.22	3.18	3.50	3.07	3.13	0.58	3.27	3.21	2.86	3.11	0.40
MMP28	222937_s_at	6.41	5.98	6.29	6.06	6.05	6.39	6.44	5.80	0.94	6.01	6.00	6.06	6.02	0.18
MMP28	224207_x_at	7.02	6.65	6.76	6.73	6.94	6.88	6.92	6.94	0.16	7.12	6.64	7.07	6.94	0.12
MMP28	239272_at	2.67	3.08	2.65	2.74	2.67	2.86	2.86	2.72	0.93	2.75	2.88	3.20	3.06	0.33
MMP28	239273_s_at	2.93	3.04	3.21	3.04	3.29	2.88	3.11	3.11	0.73	3.49	3.32	2.89	3.11	0.47
MT1P3	228016_s_at	5.42	5.90	5.24	5.45	5.45	5.79	5.91	5.32	0.59	5.53	5.51	5.46	5.25	0.67
TIMP1	201666_at	14.20	13.94	14.45	13.96	13.75	13.98	14.34	14.05	0.44	13.71	14.00	14.47	14.45	0.92
TIMP2	203167_at	9.22	9.83	9.56	9.42	9.32	9.43	9.39	9.68	0.73	9.38	10.20	9.83	9.89	**0.02**
TIMP2	224560_at	12.11	12.56	12.27	12.73	12.59	10.95	12.17	12.81	0.57	12.83	12.97	13.05	12.93	**0.03**
TIMP2	231579_s_at	13.06	13.42	13.34	13.34	13.27	13.22	13.34	13.40	0.83	13.34	13.61	13.67	13.44	**0.02**
TIMP3	201147_s_at	11.01	11.83	9.86	10.71	10.46	11.24	9.28	10.27	**0.00**	10.71	11.58	10.50	11.11	0.64
TIMP3	201148_s_at	10.80	11.77	10.26	10.55	10.59	11.26	9.50	10.36	0.05	10.84	11.58	10.57	11.00	0.36
TIMP3	201149_s_at	10.60	12.57	10.01	11.69	11.41	10.98	9.07	11.08	0.33	11.81	12.44	10.31	12.03	0.22
TIMP3	201150_s_at	12.12	12.90	11.08	12.13	12.02	11.71	10.36	11.62	0.07	12.42	12.90	12.24	12.49	0.16

Supplemental Table S5: Impact of TNF--α and TGF-β on the extracellular proteome of human cardiac fibroblasts in comparison to untreated cells. Human cardiac fibroblasts derived from four patients were incubated with or without 10 ng/ml TNF-α or 5 ng/ml TGF-β for 72 hours followed by protein extraction from culture supernatants by TCA precipitation and analysis of 500 ng tryptic peptides by tandem mass spectrometry. Shown are fold changes in comparison to proteins derived from untreated control samples as well as the group means and the p-values. Statistical analysis was performed using paired t test.

No	Swiss Prot number	Protein symbol	Protein name	TNF-α stimulation, 72h						TGF-β stimulation, 72h					
				FC #1	FC #2	FC #3	FC #4	FC mean	p-value	FC #1	FC #2	FC #3	FC #4	FC mean	p-value
**secreted cytokines**															
1	P13500	CCL2	C-C motif chemokine 2	1.92	2.21	0.50	3.08	1.93	0.32	0.52	0.97	0.32	0.72	0.63	0.11
2	P80162	CXCL6	C-X-C motif chemokine 6	3.44	1.87	3.46	4.53	3.32	**0.01**	0.39	0.57	1.04	0.58	0.64	0.09
3	P06744	G6PI	Glucose-6-phosphate isomerase	0.77	0.57	1.43	0.79	0.89	0.43	0.76	0.87	1.17	0.88	0.92	0.37
4	Q99988	GDF15	Growth/differentiation factor 15	1.00	1.15	1.19	0.98	1.08	0.24	0.93	1.01	0.71	1.42	1.02	0.92
5	O60565	GREM1	Gremlin-1	1.16	0.83	2.18	1.20	1.34	0.33	1.21	0.46	0.88	0.82	0.84	0.34
6	P09341	GROA	Growth-regulated alpha protein	2.95	1.43	0.51	3.58	2.12	0.33	0.63	0.69	0.30	1.16	0.69	0.19
7	P05231	IL6	Interleukin-6	7.31	2.65	1.41	3.35	3.68	**0.05**	5.53	1.68	0.88	2.71	2.70	0.14
8	P10145	IL8	Interleukin-8	4.80	1.47	0.20	4.31	2.69	0.58	2.08	0.59	0.62	1.70	1.25	0.86
**collagens**															
1	P02452	CO1A1	Collagen alpha-1(I) chain	0.84	0.90	0.46	0.74	0.73	0.11	1.06	1.34	1.29	1.17	1.22	**0.04**
2	P08123	CO1A2	Collagen alpha-2(I) chain	0.78	0.84	1.48	0.73	0.96	0.64	1.01	1.37	1.37	1.47	1.31	0.05
3	P02461	CO3A1	Collagen alpha-1(III) chain	0.60	1.55	0.72	1.02	0.97	0.68	0.72	1.36	1.03	0.92	1.01	0.90
4	P02462	CO4A1	Collagen alpha-1(IV) chain	0.63	0.70	1.52	0.87	0.93	0.54	1.36	0.63	1.77	1.70	1.36	0.40
5	P08572	CO4A2	Collagen alpha-2(IV) chain	1.14	1.07	1.63	0.87	1.18	0.37	2.24	1.38	1.20	1.72	1.64	**0.04**
6	P20908	CO5A1	Collagen alpha-1(V) chain	1.25	1.52	1.55	0.89	1.30	0.16	1.87	2.06	1.16	1.61	1.68	**0.03**
7	P05997	CO5A2	Collagen alpha-2(V) chain	1.56	1.10	1.02	0.88	1.14	0.45	2.45	1.16	1.53	1.82	1.74	**0.05**
8	P12109	CO6A1	Collagen alpha-1(VI) chain	0.73	1.10	1.12	0.81	0.94	0.50	0.91	1.17	1.07	0.83	0.99	0.87
9	P12110	CO6A2	Collagen alpha-2(VI) chain	0.70	1.29	1.22	0.72	0.98	0.75	1.22	1.27	1.17	0.90	1.14	0.20
10	P12111	CO6A3	Collagen alpha-3(VI) chain	0.76	1.29	1.38	0.96	1.10	0.66	1.12	1.40	1.34	0.74	1.15	0.51
11	Q99715	COCA1	Collagen alpha-1(XII) chain	0.85	0.97	1.04	0.82	0.92	0.21	0.67	0.97	0.98	1.27	0.97	0.71
**MMPs and TIMPs**															

1	P03956	MMP1	Interstitial collagenase	0.95	1.05	0.58	1.08	0.91	0.48	1.08	0.68	0.37	1.22	0.84	0.38
2	P08253	MMP2	72 kDa type IV collagenase	0.99	1.52	0.70	1.07	1.07	0.87	1.19	1.18	1.51	1.11	1.25	0.05
3	P08254	MMP3	Stromelysin-1	0.26	1.37	0.33	1.26	0.80	0.35	3.51	0.88	1.70	0.74	1.71	0.41
4	P01033	TIMP1	Metalloproteinase inhibitor 1	0.92	1.51	0.79	0.67	0.97	0.69	2.49	1.42	1.43	1.00	1.59	0.12
5	P16035	TIMP2	Metalloproteinase inhibitor 2	0.43	0.96	0.90	0.60	0.72	0.14	1.29	1.08	1.61	1.34	1.33	**0.05**
**other secreted ECM proteins**															
1	P02751	FINC	Fibronectin	0.84	1.37	1.02	0.81	1.01	0.90	0.93	1.43	1.11	0.91	1.10	0.53
2	Q15063	POSTN	Periostin	0.80	0.76	0.84	0.56	0.74	**0.04**	0.76	1.01	0.92	0.99	0.92	0.26
3	Q15582	BGH3	Transforming growth factor-beta-induced protein ig-h3	1.29	1.19	0.85	0.62	0.99	0.77	2.38	1.20	1.18	2.45	1.81	0.08
4	P07996	TSP1	Thrombospondin-1	0.84	1.14	0.52	0.55	0.76	0.18	1.03	1.07	1.05	0.77	0.98	0.72
5	P09382	LEG1	Galectin-1	0.61	0.63	0.27	0.83	0.58	0.08	0.93	0.76	1.05	0.96	0.93	0.32
6	P98160	PGBM	Basement membrane-specific heparan sulfate proteoglycan core protein	0.95	1.13	1.25	0.98	1.08	0.37	1.10	1.02	1.07	0.74	0.98	0.76
7	Q12805	FBLN3	EGF-containing fibulin-like extracellular matrix protein 1	0.50	1.08	0.85	0.61	0.76	0.16	0.54	1.20	1.28	1.01	1.01	0.84
8	P35555	FBN1	Fibrillin-1	0.96	1.12	1.21	1.03	1.08	0.25	0.84	1.09	1.06	0.94	0.98	0.72
9	Q14767	LTBP2	Latent-transforming growth factor beta-binding protein 2	0.70	0.82	0.60	0.92	0.76	0.06	2.20	1.02	1.01	1.07	1.32	0.33
10	Q08380	LG3BP	Galectin-3-binding protein	0.97	1.50	2.40	1.18	1.51	0.17	0.83	1.16	0.94	0.76	0.92	0.39
11	P11047	LAMC1	Laminin subunit gamma-1	0.76	1.10	0.82	0.70	0.85	0.16	0.76	1.04	1.20	0.60	0.90	0.43
12	Q92626	PXDN	Peroxidasin homolog	0.68	1.00	1.08	1.09	0.96	0.64	1.31	1.35	1.34	1.06	1.26	**0.03**
13	P13611	CSPG2	Versican core protein	0.89	0.75	1.84	0.72	1.05	0.89	1.90	0.67	1.70	1.32	1.40	0.34
14	Q9Y4K0	LOXL2	Lysyl oxidase homolog 2	0.99	1.08	0.55	1.03	0.91	0.48	1.66	1.02	0.98	0.83	1.12	0.64
15	P21810	PGS1	Biglycan	0.65	1.58	1.06	0.70	1.00	0.77	1.10	1.51	3.58	1.09	1.82	0.19
16	P10915	HPLN1	Hyaluronan and proteoglycan link protein 1	0.84	1.05	1.05	0.83	0.94	0.39	1.13	0.99	1.06	1.27	1.11	0.15
17	Q16610	ECM1	Extracellular matrix protein 1	0.91	1.42	0.69	0.76	0.94	0.58	1.34	1.39	1.08	1.02	1.21	0.10
18	P23142	FBLN1	Fibulin-1	0.69	1.14	0.88	0.79	0.88	0.25	0.75	1.19	1.12	0.78	0.96	0.63
19	Q76M96	CCD80	Coiled-coil domain-containing protein 80	0.53	1.02	0.97	0.41	0.73	0.18	0.49	1.09	1.10	1.37	1.01	0.82
20	P07942	LAMB1	Laminin subunit beta-1	0.76	0.87	0.94	0.62	0.79	0.08	0.76	0.98	1.26	0.62	0.90	0.44
21	P01009	A1AT	Alpha-1-antitrypsin	0.92	1.13	1.62	0.33	1.00	0.69	0.73	1.05	1.19	0.26	0.81	0.37
22	Q14112	NID2	Nidogen-2	0.70	1.40	2.61	0.61	1.33	0.76	0.64	1.40	1.18	1.02	1.06	0.92

23	Q16363	LAMA4	Laminin subunit alpha-4	0.89	0.74	1.16	0.62	0.85	0.26	0.59	0.90	1.07	0.67	0.81	0.17
24	P27797	CALR	Calreticulin	0.85	1.38	1.14	0.91	1.07	0.69	0.92	1.24	0.97	1.02	1.04	0.68
25	P29279	CTGF	Connective tissue growth factor	0.84	1.97	0.50	1.20	1.13	1.00	1.91	1.55	1.29	1.23	1.50	**0.03**
26	Q14766	LTBP1	Latent-transforming growth factor beta-binding protein 1	1.00	1.36	0.55	0.76	0.92	0.51	1.52	1.37	0.57	1.47	1.23	0.60
27	P24821	TENA	Tenascin	0.93	1.09	0.87	2.64	1.38	0.48	0.71	1.49	2.71	0.39	1.33	0.95
28	P14543	NID1	Nidogen-1	1.30	2.01	2.10	0.93	1.59	0.13	0.86	1.57	1.69	0.78	1.22	0.53
29	Q13361	MFAP5	Microfibrillar-associated protein 5	0.50	0.83	0.67	0.59	0.65	**0.02**	0.71	0.93	1.41	1.09	1.04	0.97
30	Q96CG8	CTHR1	Collagen triple helix repeat-containing protein 1	0.91	1.06	1.02	1.43	1.10	0.45	0.88	0.88	3.26	1.28	1.57	0.41
31	Q08629	TICN1	Testican-1	0.70	1.59	0.60	0.90	0.95	0.59	1.50	1.58	2.39	1.36	1.70	**0.03**
32	P13497	BMP1	Bone morphogenetic protein 1	0.79	0.96	0.42	1.00	0.79	0.25	0.88	0.94	1.06	1.06	0.99	0.73
33	O00468	AGRIN	Agrin	0.88	0.92	0.60	1.53	0.98	0.72	0.87	1.18	0.91	0.70	0.92	0.39
34	P07585	PGS2	Decorin	0.32	1.17	0.72	0.58	0.70	0.18	1.49	0.69	0.64	0.69	0.88	0.40
**other secreted proteins**															
1	P05121	PAI1	Plasminogen activator inhibitor 1	0.89	1.22	0.99	2.24	1.33	0.37	1.61	1.27	1.30	1.15	1.33	**0.03**
2	Q16270	IBP7	Insulin-like growth factor-binding protein 7	0.98	1.25	0.76	0.97	0.99	0.81	1.35	1.15	1.26	1.00	1.19	0.09
3	P01024	CO3	Complement C3	0.81	1.00	1.34	0.85	1.00	0.87	0.60	0.88	0.92	0.48	0.72	0.11
4	P02787	TRFE	Serotransferrin	0.71	1.11	0.70	0.83	0.84	0.17	0.76	1.07	0.99	0.96	0.95	0.44
5	P61769	B2MG	Beta-2-microglobulin	1.03	1.09	0.38	1.26	0.94	0.61	1.04	0.94	0.72	0.76	0.86	0.17
6	P01834	IGKC	Ig kappa chain C region	0.49	1.05	0.64	0.66	0.71	0.10	0.60	1.02	1.00	1.25	0.97	0.69
7	P08603	CFAH	Complement factor H	1.01	1.57	1.08	1.17	1.21	0.17	0.90	1.09	1.01	0.98	1.00	0.87
8	P01857	IGHG1	Ig gamma-1 chain C region	0.50	1.49	0.77	1.15	0.98	0.69	0.68	1.39	0.88	1.47	1.10	0.80
9	P02647	APOA1	Apolipoprotein A-I	0.77	1.30	1.06	0.91	1.01	0.94	0.76	1.13	0.70	1.03	0.91	0.38
10	P19823	ITIH2	Inter-alpha-trypsin inhibitor heavy chain H2	0.57	1.01	0.94	0.91	0.86	0.27	0.61	0.94	1.32	0.88	0.94	0.56
11	P07093	GDN	Glia-derived nexin	3.09	1.45	1.15	0.71	1.60	0.37	11.98	1.72	2.66	1.61	4.49	0.10
12	P01023	A2MG	Alpha-2-macroglobulin	0.58	0.89	1.65	0.79	0.98	0.68	0.57	1.00	1.24	1.18	1.00	0.82
13	P26022	PTX3	Pentraxin-related protein PTX3	0.55	0.96	4.19	1.94	1.91	0.47	0.13	0.87	0.84	0.35	0.55	0.15
14	P00738	HPT	Haptoglobin	0.45	1.20	0.61	0.86	0.78	0.24	0.61	1.21	1.10	1.18	1.03	0.96
15	P01034	CYTC	Cystatin-C	0.84	1.31	0.65	0.97	0.94	0.58	1.58	1.10	1.32	1.01	1.25	0.13

16	O00391	QSOX1	Sulfhydryl oxidase 1	1.02	1.25	1.39	0.99	1.16	0.18	0.88	0.99	1.05	1.12	1.01	0.93
17	P17936	IBP3	Insulin-like growth factor-binding protein 3	0.83	1.69	0.54	0.47	0.88	0.43	2.83	1.64	2.20	1.47	2.04	**0.02**
18	P07602	SAP	Prosaposin	0.77	1.32	1.32	1.79	1.30	0.30	0.51	0.86	0.69	0.95	0.75	0.11
19	P04004	VTNC	Vitronectin	0.60	1.06	1.86	0.71	1.06	0.87	0.58	0.93	1.22	1.06	0.95	0.61
20	P00736	C1R	Complement C1r subcomponent	0.70	1.49	1.69	0.87	1.19	0.64	0.44	1.32	2.82	0.79	1.34	0.88
21	P01876	IGHA1	Ig alpha-1 chain C region	0.61	1.17	0.69	1.34	0.95	0.63	0.86	1.06	0.83	1.00	0.94	0.34
22	Q15113	PCOC1	Procollagen C-endopeptidase enhancer 1	0.58	1.07	0.97	0.71	0.83	0.23	0.67	1.17	1.35	0.87	1.02	0.91
23	Q02818	NUCB1	Nucleobindin-1	1.20	1.68	3.86	0.88	1.90	0.23	0.96	1.23	0.65	1.01	0.96	0.67
24	O43852	CALU	Calumenin	0.62	1.09	1.57	0.75	1.01	0.80	0.65	1.05	1.31	0.89	0.98	0.73
25	O43854	EDIL3	EGF-like repeat and discoidin I-like domain-containing protein 3	0.93	0.84	1.76	0.88	1.10	0.79	1.06	0.85	1.03	1.24	1.05	0.68
26	O76061	STC2	Stanniocalcin-2	1.23	0.90	1.58	1.10	1.20	0.25	1.39	0.67	1.00	0.91	1.00	0.81
27	P15291	B4GT1	Beta-1,4-galactosyltransferase 1	1.84	1.82	0.80	1.36	1.45	0.20	1.62	1.41	0.58	1.02	1.16	0.77
28	P28300	LYOX	Protein-lysine 6-oxidase	0.73	1.36	1.26	0.59	0.98	0.73	1.74	1.20	2.16	1.14	1.56	0.07
29	P01859	IGHG2	Ig gamma-2 chain C region	0.76	1.49	0.84	0.91	1.00	0.82	0.76	1.43	0.90	0.98	1.02	0.93
30	Q12841	FSTL1	Follistatin-related protein 1	1.41	0.77	2.02	0.99	1.30	0.43	1.06	0.53	0.71	1.03	0.83	0.27
31	P02652	APOA2	Apolipoprotein A-II	0.55	0.65	1.72	0.50	0.85	0.37	0.86	0.95	0.71	0.46	0.74	0.13
32	P24592	IBP6	Insulin-like growth factor-binding protein 6	0.85	0.66	1.04	0.67	0.80	0.12	0.97	0.69	1.29	1.05	1.00	0.86
33	Q13219	PAPP1	Pappalysin-1	1.42	1.70	0.59	1.95	1.42	0.41	1.23	1.15	1.35	1.06	1.20	**0.04**
34	Q14393	GAS6	Growth arrest-specific protein 6	0.37	0.96	0.53	0.73	0.65	0.10	2.78	0.99	0.87	0.61	1.31	0.79
35	P10124	SRGN	Serglycin	1.31	1.35	0.98	1.59	1.31	0.09	0.47	0.92	1.31	0.94	0.91	0.52
36	Q13443	ADAM9	Disintegrin and metalloproteinase domain-containing protein 9	0.73	1.28	1.75	0.85	1.15	0.70	1.01	1.08	3.03	0.79	1.47	0.48
37	P07858	CATB	Cathepsin B	1.25	1.18	0.26	2.27	1.24	0.95	0.61	0.75	0.54	1.18	0.77	0.17
38	P01008	ANT3	Antithrombin-III	1.07	1.16	0.82	0.81	0.97	0.64	0.74	0.93	1.11	0.79	0.89	0.26
39	Q9UBP4	DKK3	Dickkopf-related protein 3	0.33	0.94	1.80	0.74	0.95	0.57	0.44	1.23	4.09	0.98	1.69	0.70
40	P00751	CFAB	Complement factor B	1.06	0.90	3.21	2.46	1.91	0.21	0.63	0.87	1.35	1.02	0.97	0.69
41	P08476	INHBA	Inhibin beta A chain	3.67	1.84	1.62	1.14	2.07	0.08	23.37	1.68	1.53	1.74	7.08	0.18
42	P05067	A4	Amyloid beta A4 protein	0.59	1.03	0.92	0.95	0.87	0.30	0.86	1.05	0.81	0.95	0.92	0.21
43	Q4ZHG4	FNDC1	Fibronectin type III domain-containing protein 1	0.70	0.93	4.12	1.16	1.73	0.52	0.90	1.39	2.14	1.60	1.51	0.14

44	Q92520	FAM3C	Protein FAM3C	0.64	0.77	1.60	0.55	0.89	0.44	1.49	0.92	2.59	1.04	1.51	0.26
45	P10599	THIO	Thioredoxin	0.82	1.22	0.76	3.56	1.59	0.54	0.18	0.64	1.02	0.91	0.69	0.26
46	P06396	GELS	Gelsolin	1.16	1.21	0.67	0.49	0.88	0.44	1.53	0.86	0.96	0.84	1.05	0.91
47	P55058	PLTP	Phospholipid transfer protein	0.98	0.86	2.13	1.12	1.27	0.45	2.01	0.60	1.43	1.01	1.26	0.62
48	P04083	ANXA1	Annexin A1	1.11	1.47	1.25	0.96	1.20	0.17	0.68	0.94	0.66	1.11	0.85	0.23
49	P07339	CATD	Cathepsin D	0.83	1.83	0.68	0.95	1.07	0.99	0.88	1.43	0.50	0.65	0.86	0.39
50	P10646	TFPI1	Tissue factor pathway inhibitor	1.29	1.94	0.58	0.75	1.14	0.94	1.03	1.03	0.57	1.20	0.96	0.65
51	P01860	IGHG3	Ig gamma-3 chain C region	1.10	0.68	0.20	0.75	0.68	0.24	0.79	0.55	0.76	2.31	1.10	0.84
52	P80303	NUCB2	Nucleobindin-2	1.64	1.44	2.14	0.76	1.49	0.23	1.13	1.51	0.10	1.21	0.99	0.58
53	Q6FHJ7	SFRP4	Secreted frizzled-related protein 4	2.33	1.10	0.37	0.70	1.12	0.81	4.24	0.49	0.64	1.18	1.64	0.83
54	O95965	ITGBL	Integrin beta-like protein 1	1.23	0.67	0.65	0.59	0.79	0.18	1.45	0.45	1.12	1.18	1.05	0.90

Abbreviations: FC, fold change; ECM, extracellular matrix

2. Expanded Material

Supplemental Table S6: Cardiac fibroblasts derived from patients with the following characteristics.

Characteristic			
Age [years]	59	±	3
Ejection fraction [%]	29	±	4
Non-ischemic cardiomyopathy	100%		
Female	50%		

Supplemental Table S7: Description of experiment settings for LC-ESI-MS analysis. Parameters for liquid chromatography and search parameter to identify the proteins are listed.

LC-Parameters	Settings
Buffer	A – 2 % acetonitrile in 0.1 % acetic acid<br>B – acetonitrile with 0.1 % acetic acid
LC gradient	0min-2%B-1min-5%-60min-25%-70min-40%-78min-100%-79 min-100%-99min-2%
Flow rate	300 nl/min
**Search Parameter**	**Settings**
Name of peaklist-generating software and release version (number or date)	ReadW in Sorcerer built 4.0.4 (SageN) with default parameters
Name of the search engine and release version (number or date)	Sequest 2.7 in Sorcerer built 4.0.4 (SageN)
Name of database searched and release version/date	Decoy Uniprot/Swissprot rel. 2014_02 (human) /2014_10 (bovine)
Enzyme specificity considered	Fully tryptic
# of missed cleavages permitted	Missed clevages=2
Fixed modification(s) (including residue specificity)	Carbamidomethylation at cystein
Variable modification(s) (including residue specificity)	oxidation on methionine
Mass tolerance for precursor ions	10 ppm
Mass tolerance for fragment ions	1 Da
Threshold score for accepting protein identification	Peptide false positive rate of 1 %; protein teller score > 0.9
Threshold score/E-value for accepting *individual* MS/MS Spectra	Peptide Teller false positive rate 1%
Software/method used to evaluate site assignment	No PTM reported
